# Genome Features of the Endophytic Actinobacterium *Micromonospora lupini* Strain Lupac 08: On the Process of Adaptation to an Endophytic Life Style?

**DOI:** 10.1371/journal.pone.0108522

**Published:** 2014-09-30

**Authors:** Martha E. Trujillo, Rodrigo Bacigalupe, Petar Pujic, Yasuhiro Igarashi, Patricia Benito, Raúl Riesco, Claudine Médigue, Philippe Normand

**Affiliations:** 1 Departamento de Microbiología y Genética, Edificio Departamental, Campus Miguel de Unamuno, Universidad de Salamanca, Salamanca, Spain; 2 Université Lyon 1, Université de Lyon, CNRS-UMR5557 Ecologie Microbienne, Villeurbanne, France; 3 Biotechnology Research Center, Toyama Prefectural University, Kurokawa, Imizu, Toyama, Japan; 4 Genoscope, CNRS-UMR 8030, Atelier de Génomique Comparative, Evry, France; Aarhus University, Denmark

## Abstract

Endophytic microorganisms live inside plants for at least part of their life cycle. According to their life strategies, bacterial endophytes can be classified as “obligate” or “facultative”. Reports that members of the genus *Micromonospora,* Gram-positive Actinobacteria, are normal occupants of nitrogen-fixing nodules has opened up a question as to what is the ecological role of these bacteria in interactions with nitrogen-fixing plants and whether it is in a process of adaptation from a terrestrial to a facultative endophytic life. The aim of this work was to analyse the genome sequence of *Micromonospora lupini* Lupac 08 isolated from a nitrogen fixing nodule of the legume *Lupinus angustifolius* and to identify genomic traits that provide information on this new plant-microbe interaction. The genome of *M. lupini* contains a diverse array of genes that may help its survival in soil or in plant tissues, while the high number of putative plant degrading enzyme genes identified is quite surprising since this bacterium is not considered a plant-pathogen. Functionality of several of these genes was demonstrated *in vitro*, showing that Lupac 08 degraded carboxymethylcellulose, starch and xylan. In addition, the production of chitinases detected *in vitro*, indicates that strain Lupac 08 may also confer protection to the plant. *Micromonospora* species appears as new candidates in plant-microbe interactions with an important potential in agriculture and biotechnology. The current data strongly suggests that a beneficial effect is produced on the host-plant.

## Background

For a long time, it was considered that a healthy plant was a plant without microbes within its tissues. However, this view has started to change with new approaches to allow strains to grow for a longer time upon isolation as well as the use of NGS, which has permitted the identification of several strains present in the tissues of healthy plants, in particular several actinobacteria [1], [2].

Endophytic microorganisms live inside plants for at least part of their life cycle. According to their life strategies, bacterial endophytes can be classified as “obligate” or “facultative”. Obligate endophytes are strictly dependent on the host plant for their growth and survival while facultative endophytes have a stage in their life cycle during which they exist outside host plants [3]. These endophytes originate from soil, initially infecting the host plant by colonizing, for instance, the cracks formed at points of emergence of lateral roots from where they quickly spread to the intercellular spaces in the root [4]. Thus, a series of environmental and genetic factors is presumed to have a role in enabling a specific bacterium to become endophytic [5]. Conversely, Marchetti and co-workers [6] recently showed how a pathogen can evolve in a few generations to become a symbiotic endophyte by losing specific transporters and regulators linked to pathogenesis.

*Micromonospora* is a genus of Gram-positive Actinobacteria that was first isolated from soil [7]. This bacterium has received a lot of attention during natural product screening programs, given its ability to produce a very rich array of secondary metabolites [8], [9], [10]. The distribution of members of *Micromonospora* is wide-ranging since these bacteria have been isolated from different geographical zones. In addition, its habitats are also diverse and include: soil, freshwater and marine sediments, mangrove soils, rocks, and nitrogen fixing nodules of both leguminous and actinorhizal plants [11], [12], [13]. The recent report [13] that *Micromonospora* inhabits nitrogen-fixing nodules in a systematic way, has opened up a question as to what is the potential ecological role of this bacterium in the plant and whether this bacterium is in a process of adaptation from a terrestrial to a facultative endophytic life style.

Taxonomically, *Micromonospora* belongs to the family *Micromonosporaceae* which currently contains 27 genera and includes aerobic, non-acid fast and mesophilic microorganisms. Many strains produce mycelial carotenoid pigments giving the colonies an orange to red appearance, but blue-green, brown or purple pigmented strains have also been isolated. The family *Micromonosporaceae* also harbors the genus *Salinispora*, which is widely distributed in tropical and sub-tropical marine sediments. This taxon was described as the first marine actinomycete given its inability to grow in low salinity medium. Indeed, genomic information obtained from the genomes of *Salinispora tropica* and *Salinispora arenicola* provide evidence of marine adaptation of *Salinispora* species [14]. Thus, it appears that *Salinispora* evolved from a terrestrial environment to a marine habitat. In the case of some *Micromonospora* lineages, the question is whether this bacterium has followed a comparable evolution process, changing from a terrestrial to an endophytic lifestyle.

Further examples of closely related actinobacteria with different lifestyles reflected in their genomes include, among others, the genera *Frankia*, *Mycobacterium* and *Streptomyces*. In the case of *Frankia*, comparative genomic analysis of three representative strains, differing by less than 2% in their 16S rRNA genes revealed significant differences in their genome sizes (5.4–9.0 Mb) suggesting that these differences (e.g. gene deletion, acquisition and duplication, etc.) reflect their rapid adaptation to contrasted host plants and to their environments [15]. Similarly, several mycobacterial genomes were analyzed both at the nucleotide and protein levels. One of the most striking features was lipid metabolism genes with marked expansions of the number of genes related to saturated fatty acid metabolism in the pathogenic mycobacteria compared to the soil-dwelling strains [16].

In an effort to identify the genomic traits which make possible adaptation from a soil dwelling way of life to an endophytic habitat, the aim of this work was to present the genome sequence analysis of a representative strain, *Micromonospora lupini* Lupac 08, isolated from a nitrogen fixing nodule of the legume *Lupinus angustifolius*. This strain is part of a collection of more than 2000 strains isolated from nitrogen fixing root nodules of diverse legume [17], [18] and actinorhizal species [13]. Strain Lupac 08 was selected as it showed good plant growth promotion, was used previously for in situ localization studies *in planta* [11] and produced several new secondary metabolites [9], [10]. The results presented here show that the genome of *M. lupini* Lupac 08 contains a diverse array of genes that may help its survival in soils or in plant tissues, while the high number of putative plant degrading enzyme genes identified in its genome is quite surprising since this bacterium is not considered a plant-pathogen and may instead reflect their ability to bind to plant structural compounds.

## Results

### Phylogenetic position of *M. lupini* Lupac 08 and general genome features

**Figure 1:**
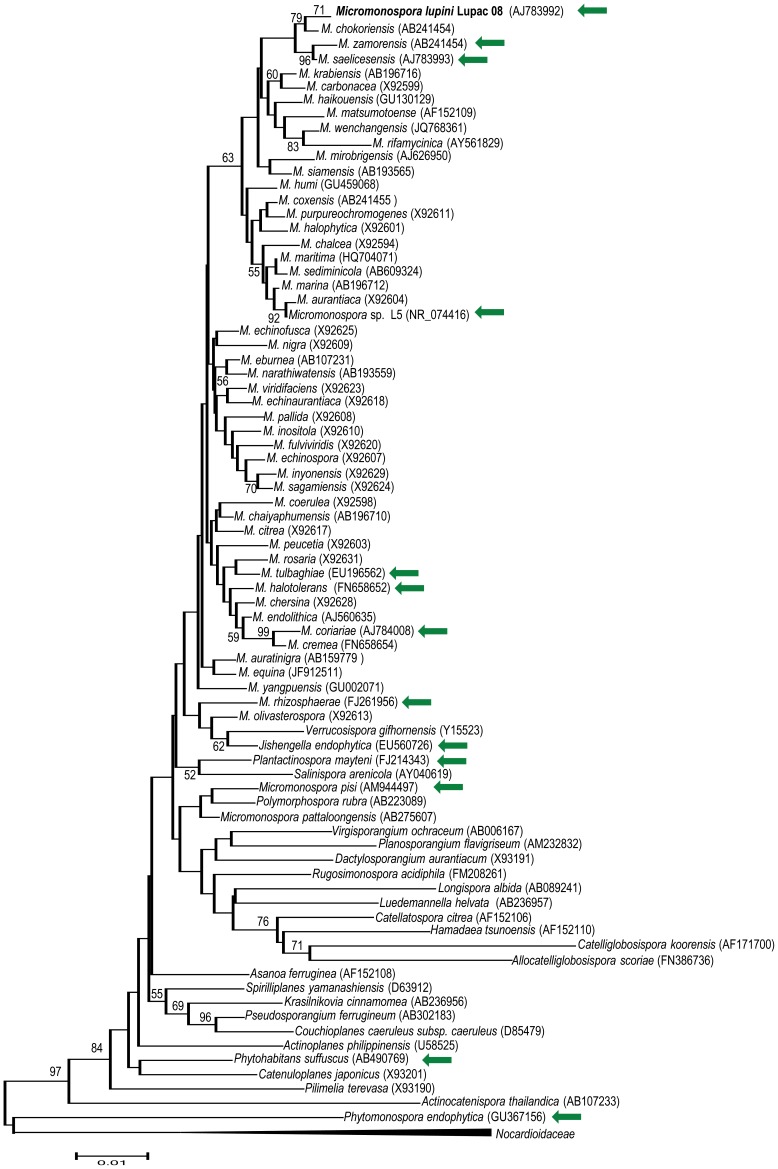
. **Neighbour-joining tree based on 16S rRNA gene sequences showing the relationship of** ***Micromonospora*** **species and other members of the family** ***Micromonosporaceae*** **.** Strains isolated from plant related sources are indicated by a green arrow.

The phylogenetic position based on 16S rRNA gene sequence analysis of strain Lupac 08 with respect to currently described *Micromonospora* species and other members of the family *Micromonosporaceae* is presented in Figure 1. Those strains associated with plant/rhizosphere sources are highlighted. Strain Lupac 08 was clearly positioned within the genus *Micromonospora* and forms a subgroup together with the species *Micromonospora saelicesensis, Micromonospora zamorensis* and *Micromonospora chokoriensis*. These strains were isolated from a nitrogen fixing nodule, the rhizosphere of a *Pisum sativum* plant and a sandy soil, respectively. Nevertheless, a clear picture based on the habitat cannot emerge from this analysis.

**Figure 2:**
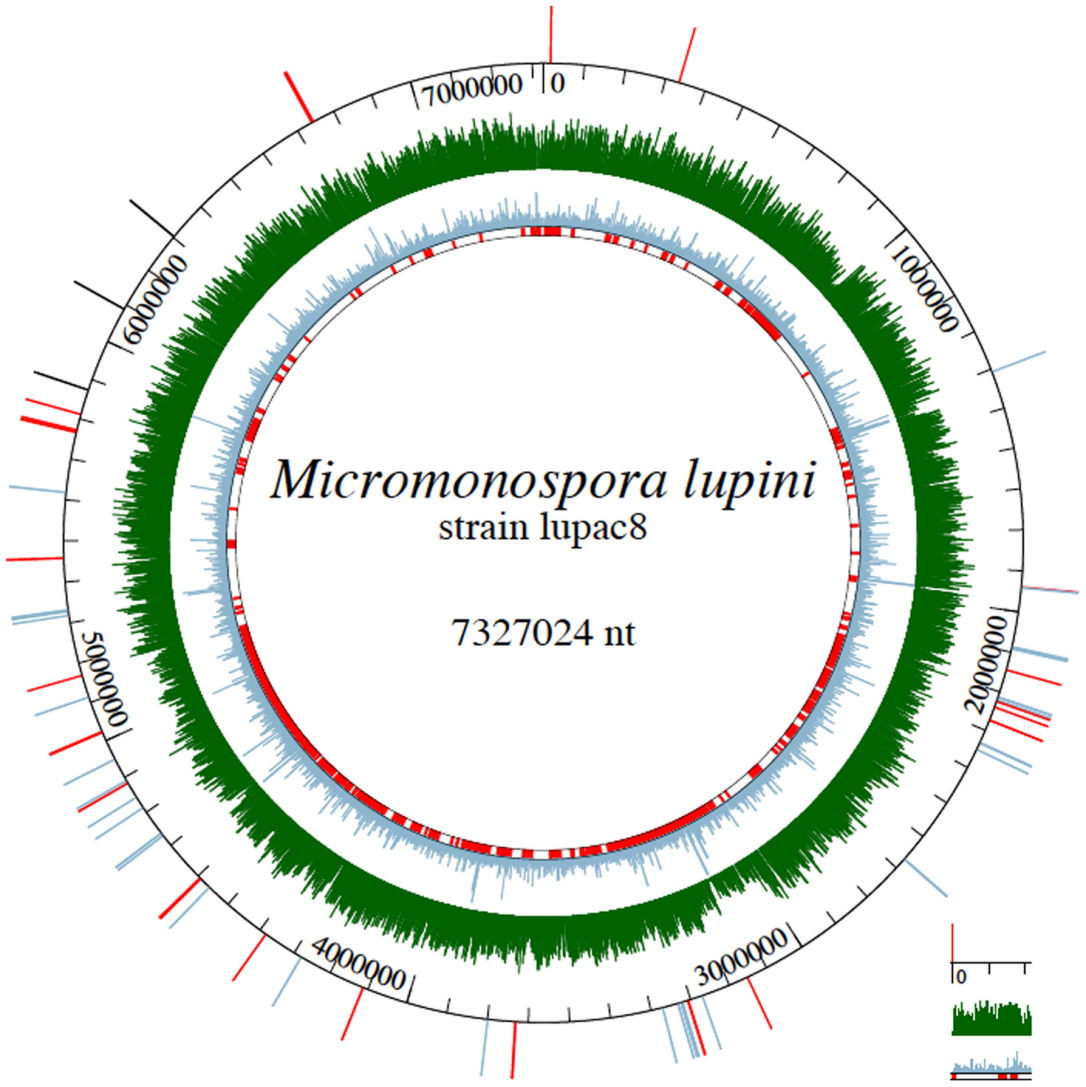
Circular representation of *Micromonospora lupini* Lupac 08. Circles displayed from the outside in: 1. Cellulose-binding genes in black, chitin-binding genes in red, lectin genes in lavender blue; 2. Genome coordinates; 3. MW; 4. GC% (linear range between 65 and 80%); 5. Regions of genome plasticity according to the RGP_Finder method (Mage platform) based on synteny breaks between the query genome (Lupac 08) and close genomes (*Micromonospora aurantiaca* ATCC 27029^T^, *Micromonospora* sp. L5 and *Verrucosispora maris* AB-18-032^T^) correlated with HGT features (tRNA hotspot, DNA repeats, mobility genes), and compositional bias and GC deviation computation. C1 to C15 indicate the position of the 15 clusters of genes coding for secondary metabolites of Table 4.

*M. lupini* Lupac 08 was shown to have a circular chromosome of 7,327,024 bp with a GC content of 71.96% and no plasmid. A total of 7158 genomic objects were identified: 7,054 protein-coding, 10 rRNAs, 77 tRNAs, and 12 miscRNAs genes. The average gene length was 964 bp with an average intergenic distance of 126 bp. After manual validation of the automatic annotation, 61.5% (4338 CDSs) of the genes were assigned a biological function while 38.5% were registered as open reading frames (ORFs) with an unknown function. Based on the G+C skew analysis and position of *dnaA*, the probable origin of replication (*oriC*), was mapped close to the ribosomal protein *rpmH*. A circular representation of the *M. lupini* chromosome is provided in Figure 2 indicating some of the features described above.

The genomic characteristics of strain Lupac 08 and three additional *Micromonospora* genomes deposited in the public databases including *Micromonospora* sp. strain L5 isolated from root nodules of *Casuarina equisetifolia* [19] *; M. aurantiaca* ATCC 27029^T^ and *Micromonospora* sp. ATCC 39149 isolated from soil (Table 1) were compared. An important difference between the four strains was the number of tRNAs identified. *M. lupini* 08 contained by far the highest number with 77 tRNAs while the other strains had between 51 and 53. At present, *M. lupini* Lupac 08 contains one of the largest numbers of tRNAs reported among the actinobacteria sequenced. The number of rRNA and tRNA genes in a genome can be seen as an indication of positive selection. A high number of rRNA genes increases ribosome synthesis, which in turn increases the protein synthesis rate [20] and growth rate [21].

**Table 1:** Comparative genomic characteristics of *M. lupini* Lupac 08 and three *Micromonospora* genomes publicly available.

Feature	*M. lupini* Lupac 08	*M. aurantiaca* ATCC 27029^T^	*Micromonospora* sp. L5	*Micromonospora* sp. ATCC 39149
Size (Mb)	7.3	7.0	6.9	6.8
GC%	72	73	73	72
rRNA Operon	10	9	9	6
tRNA	77	52	53	51
CDS number	7054	6676	6617	5633
Average gene size (kb)	946	964	969	975
Protein-coding density (%)	90.1	90.4	90.4	89.9
Genes in COGs (%)	70.2%	68.3%	69%	nd

nd, not determined.

### Comparative genome analysis

#### COG distribution

Nearly 70% of the CDS were classified into clusters of orthologous groups (COGs, Table S1). Thus, 4873 out of 7054 CDS were assigned to 24 different categories, including those for amino acid transport and metabolism (E, 12.7%), transcription (K, 10.8%), carbohydrate transport and metabolism (G, 9.7%), inorganic ion transport and metabolism (P, 8.7%), energy production and conversion (C, 5.5%), and signal transduction mechanisms (5.5%).

The COG distribution of *M. lupini* was similar to that observed in other bacteria in the family *Micromonosporaceae*, however various differences were detected such as the abundance of genes related to carbohydrate transport and metabolism. Among the *Micromonospora* genomes currently available, *M. lupini* Lupac 08 contained the highest percentage of genes (9.7%, 685) related to this category, followed by *Micromonospora* sp. L5 (8.9%, 598) and *M. aurantiaca* ATCC 27029 (8.5%, 576). The gene contents (in the same COG category) of other bacterial genomes classified in the family *Micromonosporaceae* were lower as in the case of *S. tropica* CNS-205 (7.4%, 391) and *S. arenicola* CNH-643 (6.4%, 374) two obligate marine actinomycetes. On the other hand, the overall COG profiles of *Verrucosispora maris* AB-18-032^T^ (genome size 6.7 Mb) and *M. lupini* Lupac 08 were very similar and no clear differences were found. Although *V. maris* was isolated from a sea sediment, it does not require sea salts for growth and it is not considered an obligate marine microorganism unlike *S. tropica* and *S. arenicola*. Thus, its metabolism suggests a terrestrial life style. Micromonosporae are well known for their ability to degrade complex polysaccharides such as cellulose, chitin and lignin [22], [23]. In particular, cellulose is frequently utilized as a carbon source [24], [25]. Therefore the abundance of these genes in the genome of strain Lupac 08, at first glance may not seem surprising, however, the value of 9.7% is comparable to that of highly active cellulolytic microorganisms such as *Cellulomonas flavigena* 134^T^ (9.5%) and *Thermobifida fusca* XY (7.9%), which are abundant in cellulose enriched environments such as soil, or plant tissues.

#### Synteny

**Figure 3:**
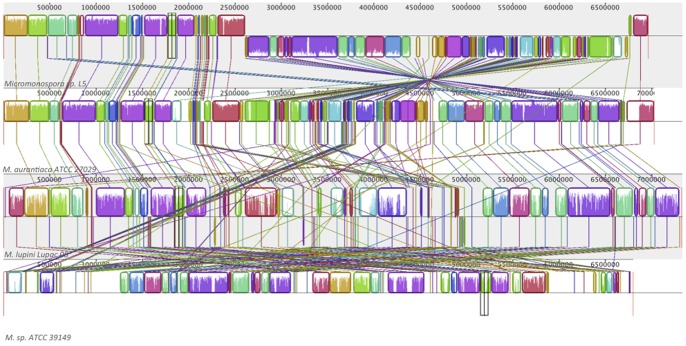
MAUVE alignment of the genome sequences of *Micromonospora lupini* Lupac 08, *Micromonospora* sp. L5, *Micromonospora aurantiaca* ATCC 27029^T^ and *Micromonospora* sp. ATCC 39149. When boxes have the same colour, this indicates syntenic regions. Boxes below the horizontal line indicate inverted regions. Rearrangements are shown by coloured lines. Scale is in nucleotides.

The genome sequence of strain Lupac 08 was aligned with those of *Micromonospora* sp. L5, *M. aurantiaca* ATCC 27029^T^ and *Micromonospora* sp. ATCC 39149^T^ (Fig. 3). Although the four genomes share a significant amount of genetic characteristics, they have undergone various inversions and translocations and *M. lupini* Lupac 08 contains the highest number of non-conserved regions. In addition, this alignment shows a high homology between strains *Micromonospora* sp. L5 and *M. aurantiaca* ATCC 27029^T^ confirming their close phylogenetic relationship as suggested by 16S rRNA gene phylogeny (Fig. 1); nevertheless, strain L5 shows a large inversion event. Thus, although the four *Micromonospora* genomes share many common features, it is also evident that *M. lupini* contains unique genomic regions as compared to *M. aurantiaca* ATCC 27029^T^ or *Micromonospora* sp. L5.

### Diversity of *Micromonosporae*: core vs. flexible gene pool

**Figure 4:**
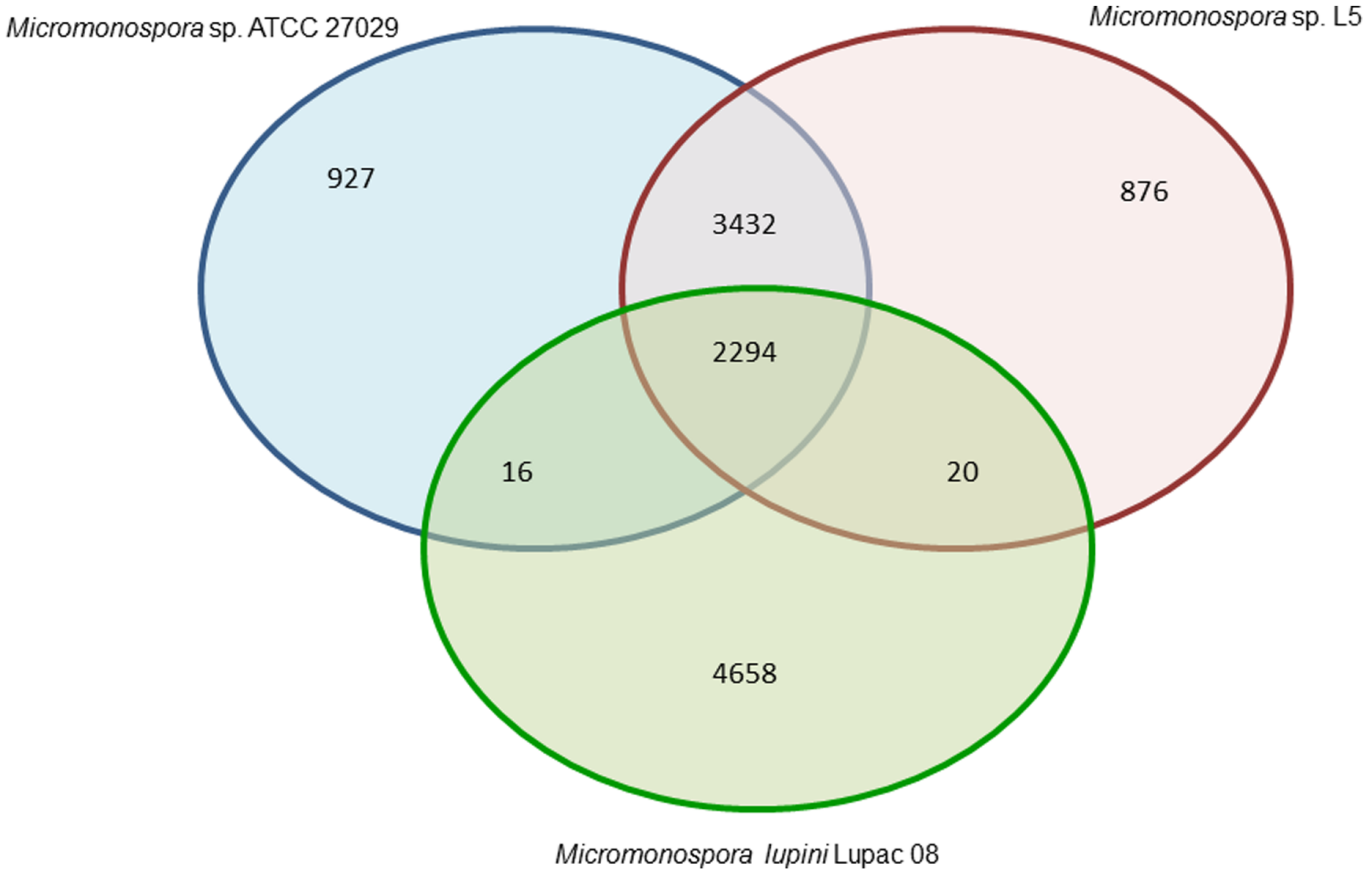
Venn diagram showing the number of clusters of orthologous genes, shared and unique, between *M. lupini* Lupac 08, *Micromonospora* sp. L5 and *M. aurantiaca* ATCC 27029^T^.

Using the *Micromonospora* genomes of strains M. *lupini* Lupac 08, *M. aurantiaca* ATCC 27029^T^ and that of *Micromonospora* sp. L5 available in the NCBI [19], the core genome was calculated using the SiLix software [26]. The core genome was composed of 2294 CDSs, which correspond to approximately 32% of the predicted proteome. In addition, *M. lupini* Lupac 08 contained the highest number of strain specific CDSs, 4702 (66.6%), which is a very high value when compared to *Micromonospora* sp. L5 and *M. aurantiaca* ATCC 27029^T^ (13–14%, Figure 4), which both share a high gene similarity (85–86%).

Horizontal gene transfer is universally recognized as an efficient mechanism for microorganisms to acquire functions that enable them to adapt to environments with different selective pressures. Therefore insertion elements, transposases, integrated phages, and plasmids can be related to the plasticity of a genome. Strain Lupac 08 contained 49 CDSs (0.7%, of total CDSs) related to gene exchange including eight integrases and eleven recombinases. Except for seven CDSs, most of these genes were grouped into 20 clusters. Interestingly, eight of these mobile element clusters were found near genes related to carbohydrate transport and metabolism.

#### Metabolic Features

**Figure 5:**
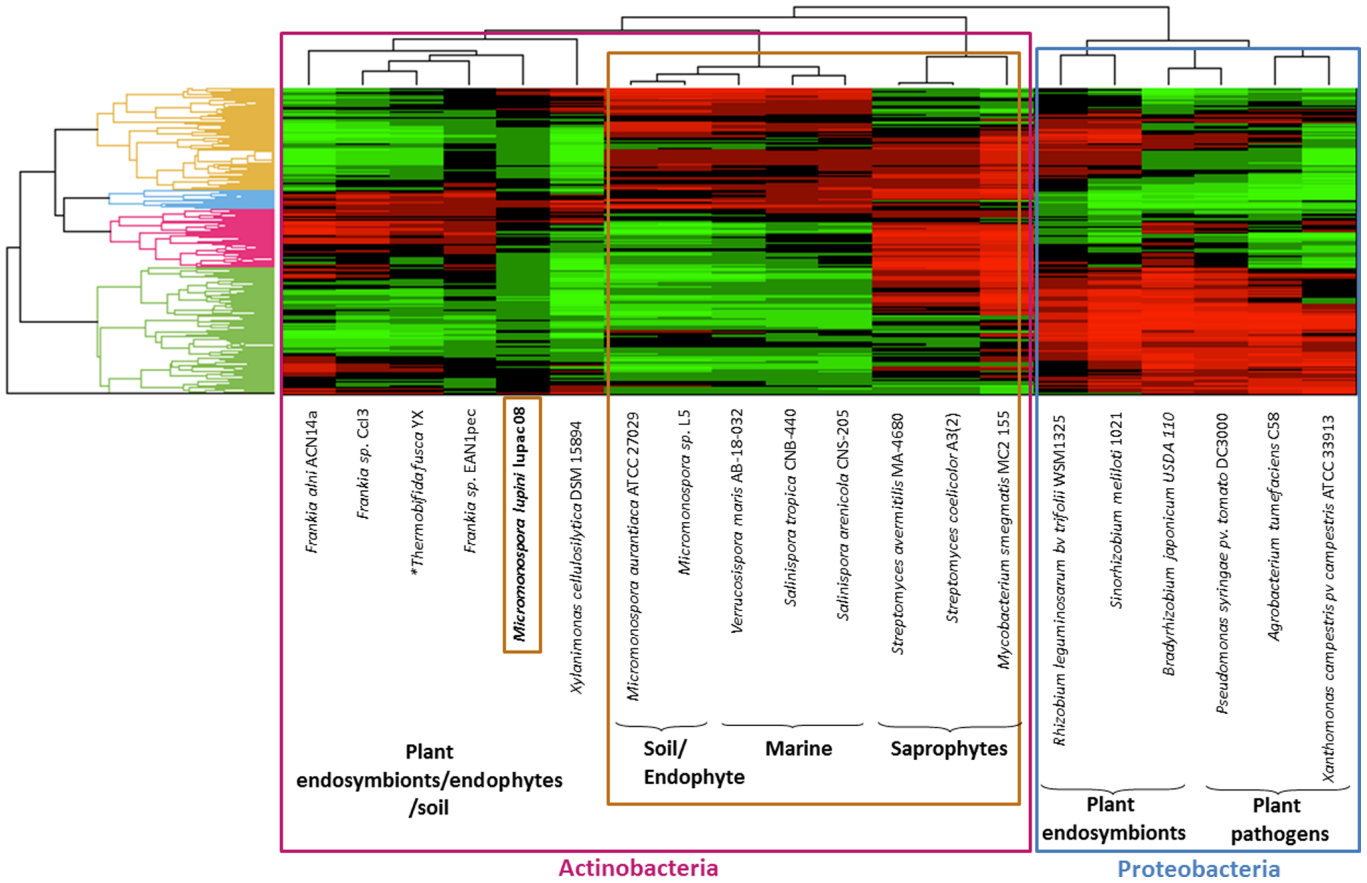
Bicluster plot of the metabolic profiles of *M lupini* Lupac 08 and 20 other bacterial genomes.

A metabolic pathway reconstruction was performed between the genome of strain Lupac 08 and 20 additional strains among which plant pathogens, symbiotic and saprophytic bacteria were included. The distribution and grouping of the microorganisms analyzed using 798 metabolic routes are presented in Figure 5. A good correlation was obtained between the microorganisms, their life style and phylogeny. Two main groups were obtained, the proteobacteria and actinobacteria. Within the actinobacteria, three clusters were clearly identified: the first one contained strains that belonged to the family *Micromonosporaceae*, the second cluster corresponded to various streptomycetes and the third cluster included the three *Frankia* genomes. Surprisingly, *Micromonospora lupini* Lupac 08 showed a closer metabolic relationship with the three *Frankia* strains (ACN14a, CcI3 and EAN1pec) than with the other two *Micromonospora* genomes.

### Plant/Soil-associated life style

#### Transport systems

Organisms living in endophytic associations need to share resources with their host. Membrane transport systems play essential roles in cellular metabolism and activities. Current data suggest a correlation of transporter profiles to both evolutionary history and the overall physiology and lifestyles of organisms [27].

A total of 631 CDSs were located in the genome of *M. lupini* coding for a large diversity of transporters, representing approximately 8.9% of the genome. The majority of CDSs were related to ATP-binding (dependent) transporters of which 362 corresponded to ABC transporters; the next most abundant (215 CDSs) coded for secondary transporters, with 105 classified in the Major Facilitator Superfamily –MFS-); 17 transporters belonged to ion channels and 20 were unclassified. The number of transporters determined in *M. aurantiaca* ATCC 27029^T^ and *Micromonospora* sp. L5 were lower with 575 and 587, respectively (Table 2).

**Table 2:** Transporters identified in the genome of *M. lupini* Lupac 08 and comparison with other bacteria with a plant/soil associated life styles.

	*M. lupini* Lupac 08	*M. aurantiaca* ATCC 27029^T^	*Micromonospora* sp. L5	*S. arenicola* CNS 205	*S. tropica* CNB 440	*F. alni* ACN14	*Frankia* sp. CcI3	*F. symbiont Dastica glomerata*	*B. japonicum* USDA6^T^	*R. leguminosarum Bv trifolii WSM 3125*	*Enterobacter* sp. 368	*S. coelicolor A32*	*S. scabiei* 8722	*Pseudomonas syringae pv phaseolicola 1448A*
Genome size (Mb)	7.3	7.0	6.9	5.7	5.2	7.5	5.4	5.3	9.6	7.4	4.6	9.1	10	5.9
Total transport proteins	631	575	587	405	413	433	253	300	1138	1087	662	798	775	670
Transporters (%)	8.9	8.5	8.7	7.1	7.8	6.4	4.5	7.1	11.8	15.5	15.6	9.7	8.2	12.5
No. Transporters/Mb genome	0.08	0.08	0.08	0.07	0.08	0.06	0.05	0.06	0.12	0.15	0.14	0.09	0.08	0.11
**ATP dependent (% of total)**	**379 (60.1%)**	**362 (63.0%)**	**366 (62.4%)**	**244 (60.2%)**	**247 (59.8%)**	**281 (64.9%)**	**146 (57.7%)**	**210 (70%)**	**684 (60.1%)**	**800 (73.6%)**	**317 (49.7%)**	**461 (57.8%)**	**480 (61.9%)**	**392 (58.5%)**
ABC family*	362 (95.5%)	341 (94%)	342 (93.4%)	225 (92.2%)	228 (92.3%)	262 (93.2%)	127 (87%)	189 (90%)	645 (94.3%)	769 (96.1%)	287 (90.5%)	433 (93.9%)	455 (94.8%)	346 (88.3%)
**Ion channels (% of total)**	**17 (2.7%)**	**14 (2.4%)**	**15 (2.6%)**	**9 (2.2%)**	**11 (2.7%)**	**12 (2.8%)**	**7 (2.8%)**	**7 (2.3%)**	**24 (2.1%)**	**26 (2.4%)**	**23 (3.5%)**	**19 (2.4%)**	**22 (2.8%)**	**32 (4.8%)**
**Phosphotransferase system (PTS)**	–	–	–	**4 (1%)**	–	–	–	–	**4 (0.4%)**	**6 (0.6%)**	**47 (7.1%)**	**10 (1.3%)**	**6 (0.8%)**	**5 (0.7%)**
**Secondary transporter**	**215 (34.1%)**	**185 (32.3%)**	**192 (32.7%)**	**9 (2.2%)**	**145 (35.1%)**	**130 (30%)**	**89 (35.2%)**	**75 (25%)**	**408 (35.9%)**	**234 (21.5%)**	**256 (38.7%)**	**286 (35.8%)**	**252 (32.5%)**	**226 (33.7%)**
MFS family*	105 (48.8%)	68 (36.8%	70 (36.5%)	65 (46.8%)	74 (51%)	64 (49.2%)	36 (40.4%)	33 (44%)	114 (27.9%)	69 (29.5%)	84 (32.8%)	120 (42%)	111 (44%)	72 (31.9%)
RND family		6 (3.2%)	7 (3.6%)	7 (5%)	8 (5.5%)	9 (6.9%)	7 (7.9%)	7 (9.3%)	31 (7.6%)	13 (5.6%)	18 (7%)	15 (5.2%)	18 (7.1%)	16 (7.1%)
**Unclassified**	**20 (3%)**	**13 (2.3%)**	**13 (2.2%)**	**8 (2%)**	**9 (2.2%)**	**10 (2.3%)**	**11 (4.3%)**	**8 (2.7%)**	**11 (1%)**	**16 (1.5%)**	**14 (2.1%)**	**21 (2.6%)**	**14 (1.8%)**	**11 (1.6%)**

*Number and percentage in relation to the total number of ATP dependent and secondary transporters respectively.

The number of transporters identified in the genome of *M. lupini* Lupac 08 is correlated with those in other bacteria with a plant/soil associated life style, which requires an efficient nutrient uptake system to obtain nutrients produced by the host plant, in addition to those found in the rhizosphere and the soil (Table 2) [27], [28]. However, the number of transporters identified in strain Lupac 08 was lower than those present in other bacteria such as *Bradyrhizobium japonicum* USDA6^T^ (1138, 11.8%), *Mesorhizobium loti* MAF303099 (968, 14.2%), *Sinorhizobium meliloti* Sm1021 (1024, 16.4%) and *Rhizobium leguminosarium* bv. *trifolii* WSM 3125 (1087, 15.5%), which form a very close interaction with legumes. Nevertheless, the overall distribution (types) and the percentages of these values were similar. An additional difference was the absence of phosphotransferase system transporters (PTS) in *M. lupini* as compared to the strains mentioned above and other soil/plant bacteria included in Table 2. On the other hand, the overall profile of *M. lupini* Lupac 08 was very similar to those of *Frankia* sp. ACN14a and *Frankia* sp. CcI3 which also lack a PTS system.

#### Secretion systems

Secreted proteins play a number of essential roles in bacteria, including the colonization of niches and host–pathogen interactions. In Gram-positive bacteria, the majority of proteins are exported out of the cytosol by the conserved Sec translocase system or, alternately, by the twin-arginine translocation system. In addition, a unique protein export system, the type VII or ESX secretion system also exists in some Gram positive bacteria [29].

The genome of *M. lupini* Lupac 08 encodes for 537 (7.6%) secreted proteins including several protein secretion systems (Table 3). All genes related to the Sec-dependent pathway were located and included the SecY and SecE proteins which form the membrane channel and interact with the cytoplasmic membrane protein SecG; the auxiliary proteins SecD, YajC and the ATPase SecA. In addition, the heterodimer Ffh-FtsY (MiLup08_41486 and Milup08_41460) was also present. As in other Gram-positive bacteria, *M. lupini* Lupac 08 lacks homologs of SecB, the chaperone that targets proteins to the Sec translocon for passage through the cytoplasmic membrane [30].

**Table 3:** Secretion system genes present in the genome of *M. lupini* Lupac 08.

Secretion System	Gene (Milup08_X)	Product
**Sec-dependent**	*secY* (*prlA*)(46297)	Preprotein translocase, membrane component
	*secE* (46336)	Preprotein translocase subunit secE
	*secG* (44961)	Preprotein translocase SecG subunit
	secD (42464)	Protein-export membrane protein secD
	secF (42465)	Protein-export membrane protein secF
	*yajC* (42463)	Preprotein translocase, YajC subunit
	*secA* (41087)	Protein translocase subunit secA
	*ffh* (41468)	Signal recognition particle protein
	*scRNA* (misc_RNA-12)	SRP, Ribosome-nascent chain complex (RNC)
	*yidC* (30220)	Cytoplasmic insertase into membrane protein
	*yidC*-like (43138)	Membrane protein insertase, YidC/Oxa1 family
	*yidC* (45964)	Inner membrane protein translocase component YidC
	Milup_08_41485	Signal peptidase I
	Milup_08_41486	Signal peptidase I
	Milup_08_42560	Conserved protein of unknown fuction (probable signal peptidase I)
	*lspA* (45113)	Lipoprotein signal peptidase
	*lgt (45071)*	Prolipoprotein diacylglyceryltransferase
**TAT-**	*tatA* (43424)	Sec-independent protein translocase protein tatA/E homolog
	*tatC* (43425)	Sec-independent protein translocase protein tatC homolog
**Type II- (T2SS)**	Milup_08_40403	Similar to uncharacterized protein from *Frankia* symbiont of *Diastica glomerata*
	Milup_08_40405	Putative helicase/secretion neighbourhood TadE-like protein
	*tadE* (40223)	TadE Family protein
	*tadE* (40224)	TadE Family protein
	*tadE* (42690)	Similar to TadE family protein
	*tadE* (42691)	Similar to TadE family protein
	Milup_08_40226	Type II secretion system protein
	Milup_08_40227	Type II secretion system protein
	Milup_08_40228	Type II secretion system protein E
	Milup_08_40398	Type II secretion system protein E
	Milup_08_40399	Similar to Type II secretion system protein E
	Milup_08_40401	Similar to Type II secretion system protein
	Milup_08_42693	Type II secretion system protein F
	Milup_08_42694	Type II secretion system protein F
	Milup_08_42695	Type II secretion system protein
**Type IV- (T4SS)**	Milup_08_42651	VirB4 protein-like protein
**Type VII/WXG100**-	*eccB* (40554)	ESX-4 secretion system protein eccB4
	*eccC* (40438)	FtsK/SpoIIIE family protein
	*eccC* (40557)	ESX-4 secretion system protein/cell division protein ftsK/spoIIIE
	*eccC* (46744)	FtsK/SpoIIIE-like transmembrane protein
	*eccD* (40556)	ESX-4 secretion system protein eccD4/Putative secretion protein snm4
	*eccD* (46743)	FtsK/SpoIIIE family protein
	*eccE* (40555)	Putative uncharacterized protein
	*esxA* (40381)	Putative uncharacterized protein
	*esxA* (40559)	Putative uncharacterized protein
	*esxB* (40380)	Putative uncharacterized protein
	*esxB* (40558)	Putative uncharacterized protein
	*mycP* (40382)	Peptidase S8 and S53 subtilisin kexin sedolisin
	*mycP* (40560)	Peptidase S8 and S53 subtilisin kexin sedolisin
	*mycP* (40564)	Peptidase S8 and S53 subtilisin kexin sedolisin
	*mycP* (46745)	Peptidase S8 and S53 subtilisin kexin sedolisin

TAT, twin-arginine translocation; X, corresponds to the annotation gene numbers given in parenthesis.

Genes related to the Sec-independent twin-arginine translocation pathyway (TAT), which exports prefolded proteins across the cytoplasmic membrane using the transmembrane proton gradient as the main driving force for translocation were also located in strain Lupac 08 (Table 3). Homologs of TatA and TatC were identified, however no homolog for TatB was found. Similar to other actinobacteria (e.g. *Frankia* sp. ACN14a) the *tatA* gene was found next to *tatC*. Only an ORF encoding TatC was located in the genomes of *Micromonospora* sp. L5 and *M. aurantiaca* ATCC 27029^T^ while no copies of *tatA* or *tatB* were found.

A set of fifteen genes identified as part of the type VII secretion system were located in *M. lupini* Lupac 08 (Table 3). These are arranged in three different clusters and included the essential proteins for secretion EccC, EccD, EsxA and EsxB [31]. The first cluster contains eight genes: *eccC, esxA, esxB, eccD, eccB, eccE* and two copies of *mycP*, a subtilisin-like serine protease which also appears essential but the function of which is not yet known [32]. The second cluster includes a copy of *esxA* (MiLup08_40381), *esxB* (MiLup08_40380) and *mycP*, annotated as S8 S53 subtilin kexin sedolisin (MiLup08_40382). Finally a third cluster contains the genes *eccC* (MiLup08_46744), *eccD* (MiLup08_46743) and *mycP* (MiLup08_46745).

Gram-negative bacteria use the type II secretion system to transport a large number of secreted proteins from the periplasmic space into the extracellular environment. Many of the secreted proteins are major virulence factors in plants and animals [33]. Type II secretion systems have been found in all completely sequenced plant pathogenic bacterial genomes, except in *Agrobacterium tumefaciens.* In addition, other bacteria have been shown to use secretion systems for the delivery of toxins, proteases, cellulases and lipases [34]–[37]. Genes coding for this system have also been reported for the three symbiotic strains *Frankia* [38].

Fifteen genes in *M. lupini* were annotated as components of the Type II secretion system, grouped into clusters of three to five genes (Table 3). Nine of these genes were annotated as Type II secretion system proteins including protein E and protein F; four were recorded as TadE family proteins and Milup08_40403 was annotated as an uncharacterized protein closest to one found in the *Frankia* symbiont of *Datisca glomerata*.

The secretion systems III and IV which are commonly related to plant-associated bacteria transport a wide variety of effector proteins into the extracellular medium or into the cytoplasm of eukaryotic host cells thus affecting the interaction [39]. In addition, a functional type IV system has been described in the plant symbiont *M. loti* strain R7A [40]. A gene annotated as *virB*4 and related to secretion system IV was located in Lupac 08 (MiLup08_42651), this ORF is surrounded by proteins with unknown function related to those present in the genomes of *Micromonospora* sp. L5 and *M. aurantiaca* ATCC 27029^T^.

#### Survival against plant defenses

Reactive oxygen species (ROS) play a major role in plant defense against pathogens. In response to attempted invasion, plants mount a broad range of defense responses, including the synthesis of ROS. *M. lupini* needs to survive under an oxidative environment in the rhizosphere before it can colonize plant roots and its genome revealed several genes encoding proteins to neutralize oxidative stress. The following genes were identified: three *sod* genes (MiLup08_45788, MiLup08_46012 and MiLup08_46604) that code for superoxide dismutases; a catalase HPII *katE* (MiLup08_44247); a catalase-peroxidase (*katG*, MiLup08_44435) and a catalase hydroperoxidase (*katA*, MiLup08_45857); four hydroperoxide reductases (MiLup08_40110, MiLup08_40293, MiLup08_41393, MiLup08_45407); a chloroperoxidase (MiLup08_44157) and a thiol peroxidase (MiLup08_43629).

In addition, a putative organic hydroperoxide resistance protein (Ohr, MiLup08_45098); a 4-hydroxyphenylpyruvate dioxygenase (Hpd, MiLup08_46664) and a homogentisate 1,2-dioxygenase (MiLup08_46677) were identified. Other enzymes include a glutathione peroxidase (MiLup08_45173); two glutathione transferases (MiLup08_46358 and MiLup08_41529) and four glutathione-S-transferases (*fdh*, MiLup08_42270, MiLup08_42834, MiLup08_44416 and 45648). Experimental data indicated that *M. lupini* indeed yields a catalase positive reaction [17] confirming the functionality of some of these genes. Therefore, to successfully reach the internal plant tissues, these genes may defend the bacterium against a ROS release by the plant.

### Regulation as a means of adaptation

Lifestyle can be viewed as the set of biotopes an organism can thrive into and the relationships that it establishes with other species and its abiotic components. It is one of the driving forces that contribute to the overall characteristics of bacterial genomes [41].

The *M. lupini* genome shows a strong emphasis on regulation, with 643 proteins (∼10%) predicted to have a regulatory function. This value is lower than that reported for the saprophytic strain *Streptomyces coelicolor* A3(2) with an exclusively terrestrial lifestyle (965 proteins; 12.3%) [42], but higher than the endosymbiotic strains *M. loti* MAFF303099 (542 proteins, 7.7%) [43]; *Frankia alni* ACN14a (515 proteins, 7.6%); *Frankia* sp. EAN1pec (555, 6.1%) and *Frankia* sp. CcI3 (244 proteins, 4.3%).

The genome codes for various regulator families such as TetR, AraC, LacI, ArsR, MerR, AsnC, MarR, DeoR, GntR and Crp. In addition, thirty-three ECF (extra-cytoplasmic function) sigma factors were located. Furthermore, 147 genes were related to two-component regulatory systems of which 34 were LuxR proteins. These two-component systems appear to play a crucial role in quorum sensing of Gram-positive bacteria and a positive correlation between plant-microbe interactions and the number of LuxR proteins has been suggested [44], [45].

Many regulatory genes (∼18%) were located near polysaccharide related loci including those involved in plant cell wall degradation. Specifically, 63% of cellulose degradation or cellulose binding genes had a nearby regulator (proximity ranged from 2–4 genes up or downstream). In the case of xylan metabolism, regulators were identified for 50% of the genes, while 43% of pectin metabolism genes also had a regulator nearby. An extended overview of the regulators and their associated carbohydrate genes is presented in Table S2.

### An endophytic bacterium highly equipped with an array of plant cell wall degrading enzymes

The ability of *M. lupini* lupac 08 to assimilate a wide range of sugars was previously reported [19] and this is clearly reflected in its genome. The range of simple and complex saccharides assimilated by this strain include cellobiose, cellulose, glucose, mannitol, starch, sucrose, trehalose, xylan and xylose among others. Genomic analyses confirmed the presence of a large number of genes devoted to the metabolism of carbohydrates, including many compounds of plant origin. Plant-polymer degrading enzymes such as cellulases, xylanases and pectinases have been suspected to play a role in internal plant colonization [46]. In the case of plant pathogenic bacteria and fungi, these gain access by actively degrading plant cell wall compounds using glycoside hydrolases including cellulases and endoglucanases. However, genomic analyses show that non-pathogenic endophytic microorganisms such as *Enterobacter* sp. 638 [47], *Azoarcus* BH72 [39] or the symbiotic actinobacterium *Frankia sp*. [48] have only a reduced set of cell-wall degrading enzymes.

The genome of *M. lupini* Lupac 08 revealed a significant number of genes encoding enzymes potentially involved in plant-polymer degradation but also an important number of cellulose-binding related genes. Overall, about 10% of the genome coded for genes related to carbohydrate metabolism of which 192 had a hydrolytic function. At least 79 genes putatively involved in interactions with plants and with the potential to hydrolyze plant polymers were identified (Table S1). These genes were placed into the glycosyl hydrolase families GH5, GH6, GH9, GH10, GH11, GH18, GH20, GH43, GH44 and GH62, or into the carbohydrate binding modules CBM2, CBM13, CBM33, CBM3, CBM46, CBM42, CBM5, CBM4, CBM6 and CBM32. The CBM2 family was the most abundant appearing in 46 of the 79 genes identified.

Fourteen genes were further identified as lectins or proteins with lectin binding domains, which presumably bind to and interact with carbohydrates. Some of these loci (e.g. Milup_42969, Milup_42975, Milup_44484, and Milup_44962) appear to be related to cellulases and xylanases, respectively. These proteins are important as they serve as a means of attachment between a bacterium and its host (animal or plant) and are produced by either of the two interacting organisms [48].

Compared to the 45 enzymes predicted to act on oligo- and/or polysaccharides reported for *T. fusca* XY [49], the number of these enzymes present in the genome of *M. lupini* is significantly higher.

#### Cellulose metabolism

Aerobic cellulolytic actinobacteria have been shown to use a system for cellulose degradation consisting of sets of soluble cellulases and hemicellulases. Most of these independent cellulolytic enzymes contain one or more carbohydrate binding domains [50].

A total of 46 genes were found to present a hydrolytic or binding fuction towards cellulose (Table S1). Several endoglucanases were detected in strain Lupac 08 (e.g. C1, C2 C10 and C14), these enzymes hydrolyze internal bonds at random positions of amorphous regions of cellulose and generate chain ends for the processive action of cellobiohydrolases (exoglucanases). A copy of the exoglucanase gene *cbhA* (C16) was also located in the genome. Exoglucanases act on the ends of cellulose polysaccharide chains, liberating cellobiose as the major product. β-D-glucosidases such as M108 and M109 which would further hydrolyze cellobiose were also identified. In addition, several extracellular cellulase coding genes were identified including *celA* (C3 and C6), *celB* (C5) and *celD* (C13). These results strongly suggest that strain Lupac 08 is potentially capable of completely degrading cellulose.

**Figure 6:**
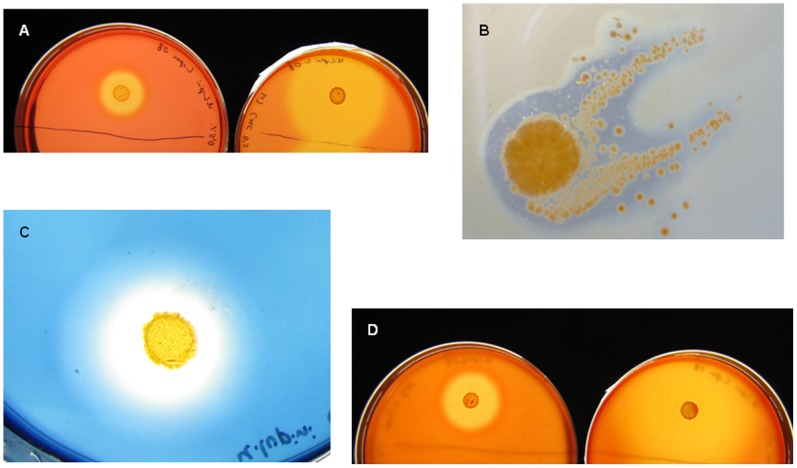
Expression of cellulose, starch, xylan and chitin degrading genes in *Micromonospora lupini* Lupac 08. (A) carboxymetheylcellulose hydrolysis at 4 (left) and 14 (right) days after inoculation. (B), starch hydrolysis at 4 days after inoculation. (C), chitin degradation at 7 days after inoculation. (D), xylan degradation at 4 (left) and 14 (right) days after inoculation.

Strain Lupac 08 was tested for *in vitro* production of cellulases. Very high cellulase activity was detected in minimal agar supplemented with carboxymethylcellulose (CMC, 0.5%) (Fig. 6A). When the culture medium was supplemented with glucose (1%) similar results were obtained indicating that this sugar did not repress nor derepress the expression of the genes responsible for the production of cellulases.

#### Hemicellulosic substrates

Genome analysis also revealed the ability of *M. lupini* to convert various hemicellulosic substrates to sugars. Twelve putative genes related to the metabolism of xylan included several copies of extracellular xylanases (X1, X3, X4, X5, X6, X7, X9, X10 and X12; see Table S1); an extracellular bifunctional xylanase/deacetylase (X8); and an arabinofuranosidase (X2) which work synergistically with xylanases to degrade xylan to its component sugars. Genes for several α-arabinofuranosidases were also identified (C17, M33, and M39); these are exo-acting enzymes which hydrolyze nonreducing arabinofuranose residues from arabinoxylan, pectins, and shorter oligosaccharides.

*In vitro* xylanase activity was detected in strain Lupac 08 when tested in a minimal medium supplemented with xylan (1%). Production of xylanases was detected after incubation for 4 days increasing significantly after 14 days (Fig. 6D). The substrate was assayed with and without glucose with similar results.

#### Starch degradation

Starch is a ubiquitous and easily accessible source of energy. In plant cells it is usually deposited as large granules in the cytoplasm. Several genes coding for amylo-α-1,6-glucosidases (e.g. M26, M32, M44, M63, M111 and M121; Table S1) were located in addition to two *amyE* homologs that code for an extracellular α-amylase. Furthermore, strain Lupac 08 was able to degrade this polymer under laboratory conditions (Fig. 6C) and it was previously shown that Lupac 08 can utilize this substrate as a carbon source [17].

#### Pectin degradation

**Figure 7:**
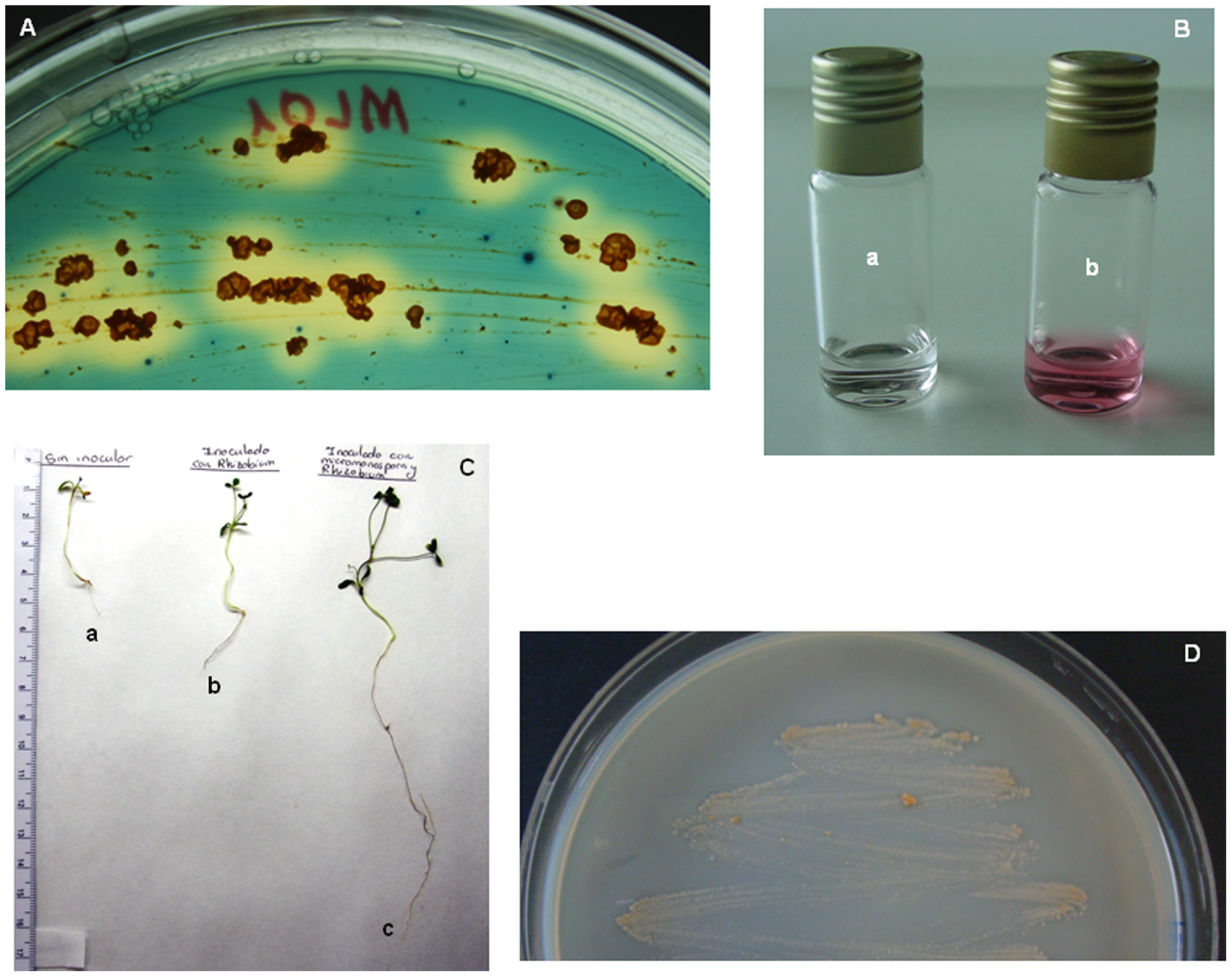
Plant growth promotion and biological control features of *M. lupini* Lupac 08. (A) Siderophore, (B) indole-3-acetic acid [a, negative control *E. coli* DH5α; b, Lupac 08] and pectinase production (D) by *M. lupini* strain Lupac 08;. (C) Plant growth promoting effect of *M. lupini* Lupac 08 on clover plantlets. a) control; b) inoculated with *Rhizobium* sp. E11; c) co-inoculated with *Rhizobium* sp. E11 and *M. lupini* Lupac 08.

Pectinolytic enzymes can degrade pectic substances either through hydrolysis (hydrolases) or trans-elimination (lyases) [51] and are important virulence mechanisms in many soft-rotting and macerating pathogens [52]. Six pectate lyases (P1, P3, P4, P5 and P6; Table S1) were located in the genome of *M. lupini*, two of which were annotated as virulence factors (P5 and P6). In addition, an extracellular pectin methylesterase gene, *pmeA*, and a gene coding for a pectate lyase involved in D-galacturonic acid hydrolysis (P7) were also identified. Interestingly, *T. fusca* XY contains two pectin lyase homologs but does not appear to possess a pectin methylesterase or a pectin acetylesterase gene. Pectinase-encoding genes are reported to be absent in other endophytic microorganisms such as *Azoarcus* sp. BH72 or *Enterobacter* sp. 638 [39], [47]. Production of pectinases was observed under laboratory conditions and activity was visualized after 8 days of incubation (Fig. 7D).

#### Expansin-like proteins

Expansins are proteins that were first described from plants [53]. These molecules function as cell wall loosening proteins by disrupting the noncovalent binding of matrix polysaccharides to cellulose [54], resulting in physical effects, such as polymer creep and stress relaxation of extended cell walls [55], [56]. Many plant-associated microorganisms including several pathogenic actinobacterial species have been shown to contain proteins with expansin-like domains [57].

Two genes (MiLup_41274 and MiLup_45306) were identified in the genome of strain Lupac 08 that encode for a secreted protein showing 42% and 48% sequence similarity to the corresponding *celA* genes of *Clavibacter michiganensis* subsp. *michiganensis* and *Clavibacter michiganensis* subsp. *sepedonicus,* respectively. This gene corresponds to a secreted β-1,4-endoglucanase (CelA) that is required for virulence and contains a C-terminal α-expansin like domain [58], [59]. In the case of *C. michiganesis* subsp. *michiganensis* CelA, this expansin-like domain is essential for development of wilting symptoms [58]. It is suggested that microbial expansins function to promote microbe-plant interactions, both harmful and beneficial ones [60].

### Plant growth promotion traits of *Micromonospora* lupini Lupac 08

Our current knowledge of plant-microbe interactions indicates that populations inhabiting a host plant are not restricted to a single microbial species but comprise several genera and species. Few reports are available regarding the presence of other microorganisms (associated or endophytic) in nitrogen fixing nodules, in spite of the fact that nodules are much richer in nutrients as compared to roots [61]. The recent reports on the isolation of large *Micromonospora* populations from nitrogen fixing nodules clearly suggest that this bacterium plays an important role which has yet to be defined.

#### Effect of *M. lupini* Lupac 08 on *Trifolium*

*Micromonospora lupini* Lupac 08 clearly produced a plant growth enhancing effect when it was co-inoculated with *Rhizobium* sp. E11 under laboratory conditions on clover plantlets. In general, the number of nodules was higher in those plants co-inoculated (18–24 nodules) with both bacteria as compared to the plants inoculated only with *Rhizobium* sp. E11 (11–15 nodules). Overall, the co-inoculated plants showed better growth and were larger in size as compared to the other two treatments (Fig. 7C). Similar results were previously observed when strain Lupac 08 was inoculated in its original host, *Lupinus* [62].

#### Nitrogen fixing capacity

Indirect evidence of nitrogen fixing genes was obtained by partial amplification of *nifH*-like gene fragments in strains *Micromonospora* sp. L5 [12] and *M. lupini* Lupac 08 [11]. In the present work the genomes of *Micromonospora lupini* Lupac 08 and *Micromonospora sp.* L5 were screened for the presence of nitrogen fixing genes to confirm this earlier finding. After thorough analysis of the complete genome, no sequences related to this biological process were detected, supporting the results reported for strain *Micromonospora* sp. L5 [19]. Nitrogenase activity detection by acetylene reduction assays carried out with strain Lupac 08 over a period of two weeks were negative. A positive result was reported for strain L5 [12.].

#### Trehalose and its role in nodulation and bacteroid survival

Trehalose is a common reserve disaccharide in the root nodules of legumes, present at high concentrations in bacteroids at the onset of nitrogen fixation [63]. It has been reported that in the interaction between *Phaseolus vulgaris* and *Rhizobium,* enhanced germination, quality and grain yield have been correlated with trehalose content, and a higher tolerance to abiotic stress [64], [65]. On the other hand, the trehalose content appears to be regulated by trehalase, a nodule stimulated plant enzyme [66], [67]. Although trehalose metabolism in leguminous plants is still poorly understood, it has been shown that in senescent nodules, trehalose becomes the most abundant nonstructural carbohydrate [68] and it is proposed that trehalose, a stress protectant accumulated in bacteria, could offset membrane injuries and/or serve as an intermediate energy reserve. Indeed, Müller *et al*. [68] showed that during terminal senescence of nodules an appreciable part of the bacteria maintained their trehalose pools and survived.

Eight genes related to the metabolism of trehalose were detected in the genome of Lupac 08; seven genes were related with trehalose synthesis (Mlup08_40949, Mlup08_43225, Mlup08_43226, Mlup_45189, Mlup_45758, Mlup08_45759 and Mlup08_45961) and one (*treA*, Mlup08_45961) with the enzyme trehalase. Barraza *et al*. [67] proposed that modification of the trehalose content in the nodules could trigger physiological alterations that would enhace carbon and nitrogen metabolism, as well as bacteroid fitness (greater survival) and nitrogen fixation, which in turn would positively impact on symbiotic interactions. *Micromonospora* may contribute to the survival of rhizobia by helping to maintain high levels of trehalose.

#### Chitin degradation and protection against pathogens

Plant β-1,3-glucanases are directly involved in defense by hydrolyzing the cell walls of fungal pathogens most commonly in combination with chitinases. Nine chitin-related ORFs were identified in *M. lupini*. Specifically, six code for a chitooligosaccharide deacetylase, several extracellular endo- and exo-chitinases and a β-N-acetyl-hexosaminidase (MiLup08_41789, MiLup08_41912, MiLup08_43481, MiLup08_44343, MiLup08_45172, MiLup08_45568), while three CDS code for putative chitin-binding domain proteins (MiLup08_41110, MiLup08_41724, MiLup08_41729). Chitinases often work synergistically with chitin-binding proteins (CBPs). The biological roles of bacterial chitinases and carbon binding proteins are easily understood in an environmental context, especially in soil (that harbour fungi and insects) and marine (shellfish) habitats and their impact on chitin cycling. However, there is an increasing amount of direct or indirect evidence suggesting that some chitinases and CBPs additionally serve as virulence factors for bacterial pathogens during infection of non-chitinous substrates [69].

Experimental data confirmed *in vitro* chitin degradation of strain Lupac 08 (Figure 6B). As with other endophytic bacteria *Micromonospora* may produce chitinases to inhibit fungal pathogens, or may produce these molecules to elicit the plant defense mechanism. Either way, it seems that *Micromonospora* would provide a benefit to its host.

### Siderophores (Iron-transport) and other secondary metabolites

Iron is an element essential for every living organism, as a cofactor of numerous proteins. Siderophores produced by plant growth promoting bacteria may reduce the growth of phytophathogens by depriving them of iron. Thus, an efficient iron uptake system can contribute to protect the host plant against pathogens. Interestingly, siderophores can also act as important virulence determinants for both plant and animal pathogens [70].

The genome of strain Lupac 08 revealed several siderophore related genes including specific iron uptake transporters, secretion of different siderophores and synthesis of siderophore receptors. Namely, a zinc/iron permease (MiLup_40258), a ferrous iron permease FTR1 (efeU, MiLup_41076) and eight iron ABC transporters (MiLup_42281-MiLup_42285). The number of the latter transporters is similar to the number of those found in the genome of the endophytic bacterium *Enterobacter* sp. 638 [47] while the plant pathogen *Erwinia amylovora* CFBP 1430 presents only three such transporters [71].

Several gene clusters related to the biosynthesis and transport of the siderophores enterobactin (MiLup_44069-MiUp_44071), aerobactin (iucA/iucC family protein, MiLup_44063 and MiLup_44064; MiLup_40326) and alcaligin (MiLup_44065) were also located. In the case of aerobactin, the gene *iucA* is highly correlated with virulence in avian pathogenic *E. coli* strains [72].

Two siderophore-interacting proteins (MiLup_40648 and MiLup_45559) were also found. One of these genes (MiLup_40648) was located next to a siderophore transporter of the RhtX/FptX family; RhtX from *S. meliloti* 2011 and FptX from *Pseudomonas aeruginosa* appear to be single polypeptide transporters from the major facilitator family for import of siderophores as a means to import iron [73]. In addition, a thiazolinyl imide reductase involved in siderophore biosynthesis was also identified (MiLup_43551).

The genome of Lupac 08 also contained several regulators including an iron-dependent repressor (IdeR, MiLup_41668), two ferric uptake regulation proteins (MiLup_40794 and MiLup_44436) and a putative iron-regulated membrane protein which suggests that these systems are highly regulated. Production of siderophores was detected experimentally (Fig. 7A).

Actinobacteria are well known to be capable of producing a vast diversity of natural secondary metabolite compounds with applications in medicine, agriculture, and other biotechnological areas [74]. Endophytic bacteria are currently of significant interest as an untapped resource of novel bioactive small molecules because their metabolites are speculated to affect the physiological conditions of host plants including growth and disease resistance. *Micromonospora* strains are well known for their capacity to produce many secondary metabolites and *M. lupini* Lupac 08 was previously screened for the production of novel compounds with antitumoral activity and the results obtained confirmed the production of a new family of molecules named Lupinacidins A, B and C [9], [10].

Fifteen clusters involved in the biosynthesis of secondary metabolites were identified in the genome of *M. lupini* Lupac 08. These included siderophores (see above), terpenes, butyrolactones, polyketides (PKS), nonribosomal peptides (NRPS), chalcone synthases and bacteriocins (Table 4). A DNA stretch of 544 kb was estimated to code predominantly for secondary metabolites, accounting for about 7.4% of the genome. This percentage is lower than that reported for the marine actinobacterium *S. tropica* (9.9%, [75]) but it is within the range of other actinobacteria e.g. *S. coelicolor* (8.2%, [43]). Interestingly, *Frankia* strains ACN14a and EAN1pec dedicate about 5% of their genomes to natural product assembly while the potential of CcI3, which has the smallest genome of the three *Frankia* strains, has a much reduced host range and is absent from most soils is significantly smaller (∼3%) [76].

**Table 4:** Comparison of secondary metabolite clusters found in the genome of *M. lupini* Lupac 08 and other related microorganisms.

Cluster	Type	*M. lupini* Lupac 08 (Milup08_X)	*Micromonospora* sp. L5	*M. aurantiaca* ATCC 27029^T^	*Verrucosispora maris* AB-18-032	*Salinispora tropica* CNB-440	*Salinispora arenicola* CNS-205	*Streptomyces coelicolor* A3(2)
1	Terpene	40204–40210	Conserved	Conserved	Conserved	Conserved	Conserved	Conserved
2	Terpene	40306–40320	Conserved	Conserved	Conserved	Absent	Absent	Present
3	Butyrolactone	40602–40668	Absent	Absent	Absent	Conserved	Conserved	Conserved
4	Type I PKS	41995–42009	Conserved	Conserved	Conserved	Conserved	Conserved	Absent
5	Terpene	43134–43144	Conserved	Conserved	Conserved	Conserved	Conserved	Conserved
6	NRPS+PKS	43546–43581	Absent	Absent	Conserved	Conserved	Absent	Partially conserved
7	Type II PKS	43804–43844	Conserved	Conserved	Conserved	Conserved	Conserved	Partially conserved
8	Siderophore	44063–44071	Conserved	Conserved	Conserved	Conserved	Conserved	Conserved
9	NRPS-Type I PKS	44386–44405	Conserved	NRPS Absent	NRPS Absent	NRPS Absent	NRPS Absent	NRPS Absent
10	Type II PKS	44613–44624	Partially conserved	Partially conserved	Partially conserved	Partially conserved	Partially conserved	Partially conserved
11	NRPS	44684–44691	Absent	Absent	Absent	Partially conserved	Absent	Absent
12	Bacteriocin	44929–44933	Conserved	Conserved	Conserved	Conserved	Conserved	Partially conserved
13	Terpene	45087–45093	Conserved	Conserved	Conserved	Absent	Absent	Conserved
14	NRPS	45439–45446	Conserved	Conserved	Conserved	Conserved	Conserved	Conserved
15	Type III PKS	46684–46700	Conserved	Conserved	Conserved	Conserved	Conserved	Absent

PKS, polyketide synthases; NRPS, non-ribosomal peptide synthases.

Several clusters identified in the genome of *M. lupini* were also located in other genomes of phylogenetically related bacteria, especially in *S. tropica* CNB-440, *S. arenicola* CNS-205 and *V. maris* AB-18-032. Nevertheless other clusters were unique to *M lupini* (Table 4). Eight of the 15 clusters identified were located in the region between coordinates 4,000 kb and 5,000 kb of the genome, close to the terminus of replication. This area of the genome also contains a high density of genes coding for the biosynthesis of various plant cell wall degrading enzymes and several transposases.

Terpene related enzymes present in the genome of *M. lupini* are involved in the synthesis of carotenoids, sugar-binding lipids and the production of pentalenolactone type antibiotics. Similar molecules have also been predicted from the genomes of the three sequenced *Frankia* strains [77]. Various polyketide biosynthetic and non-ribosomal peptide synthase pathways were also identified specifically as PKI, PKII (2 clusters), PKIII types, NRPS (2 clusters) and hybrid PKS/NRPS clusters (2 clusters). The presence of these gene clusters suggests that *M. lupini* is capable of producing a vast diversity of secondary metabolites such as the antitumor anthraquinone derivative lupinacidins reported earlier [9], [10]. Some of these metabolites may perform specialized functions in ecological niches and recent studies have reported on the importance of PKS and NRPS molecules and their potential role in communication during root colonization [78], [79]. In addition, cluster 10 contains genes that putatively code for the production of granaticin. Granaticins are antibiotics of the benzoisochromanequinone class of aromatic polyketides, the best known member of which is actinorhodin produced by *S. coelicolor* A3(2). Production of granaticins has mainly been reported from *Streptomyces* strains [80]. NRPS cluster 11 (see Table 4), appeared to be unique to strain Lupac 08 as this group of genes was not detected in any of the other genomes compared except in *S. tropica* CNB-440 where it seems to be only partially conserved.

The PKS type III cluster corresponds to several genes that code for the production of naringenin, a central precursor of many flavonoids. It has recently been proposed that flavonoids play an important role in the establishment of plant root endosymbioses. In the case of legume-*Rhizobium* interactions, flavonoids released by plant roots induce genes involved in nodulation [81]. In a similar way it has also been suggested that these molecules play an important role during the early stages of the symbiotic association between *Frankia* and actinorhizal plants [82]. *Micromonospora* flavonoids may contribute to support communication between the nitrogen fixing bacteria and their host plants.

Genomic information also revealed that *M. lupini* has the potential to produce bacteriocins (cluster 12) as suggested by the presence of a putative short-chain dehydrogenase/reductase.

### Phytohormones

#### Indole-3 acetic acid

Diverse bacterial species have the ability to produce auxinic phytohormones such as indole-3-acetic acid (IAA) and a few can also produce phenyl-acetate (PAA) such as *Frankia alni* [83], [84]. Different biosynthesis pathways have been identified and redundancy for IAA biosynthesis is widespread among plant-associated bacteria [83]. Interactions between IAA-producing bacteria and plants may lead to several outcomes, from pathogenesis to phytostimulation [85]. The genome of *M. lupini* Lupac 08 contains a gene (Milup_45687) potentially involved in the biosynthesis of IAA via the indole-3-acetonitrile pathway. This gene corresponds to the conversion of indole-3-acetonitrile to indole-3-acetic acid. Nitrilases with specificity for indole-3-acetonitrile have been reported in *Alcaligenes faecalis* [86]. In *A. tumefaciens* and *Rhizobium* spp. nitrile hydratase and amidase activity could be identified, indicating the conversion of indole-3-acetonitrile to indole-3-acetic acid via indole-3-acetamide [87]. Analysis of IAA production by strain Lupac 08 was carried out, yielding a positive result (Fig. 7B).

#### Acetoin and 2,3-butanediol

The volatile compounds acetoin and 2,3-butanediol produced by bacteria such as *Bacillus subtilis* GB03 and *Bacillus amyloliquefaciens* IN937a have been reported as plant growth promoting hormones [88]. Several genes were located in the genome of strain Lupac 08 which could be involved in the production of these compounds. Two copies of the gene *pdhB* (MiLup_40114 and MiLup_43782) that encode the enzyme pyruvate dehydrogenase were identified. This enzyme transforms pyruvate to acetaldehyde and in this process a small fraction of pyruvate is converted to acetoin as a by-product. In addition, three acetolactate synthases involved in the synthesis of acetolactate from pyruvate are present (MiLup_41336, MiLup_41383 and MiLup_41384). Under aerobic conditions, acetolactate is converted to acetoin by the enzyme acetoin dehydrogenase (MiLup_41670).

## Discussion

The most extensively studied bacteria interacting with plants are Gram-negative proteobacteria because they are readily isolated from plant tissues and genetically handled for interaction studies. However, the impact of Gram-positive bacteria on plants should not be underestimated as has been done for many years mainly due to their slow growth. *M. lupini* Lupac 08, a Gram-positive actinobacterium was isolated from the internal root nodule tissues of *Lupinus angustifolius* but it is only a representative of a large collection of more than 2000 *Micromonospora* strains isolated from diverse legumes and actinorhizals from different geographical locations. So far, several genomes of root symbionts and soil saprophytes have been studied; therefore we decided to focus on an intermediary category, that of facultative endophytes.

Lupac 08 was isolated from lupine nodules, and shown to produce the anticancer agents Lupinadicin A, B and C. The genome of strain Lupac 08 was sequenced to obtain information about the potential ecological role of *Micromonospora* in interaction with legumes and actinorhizal plants. Genomic analysis revealed several strategies which are probably necessary to lead a successful lifestyle as a saprophyte in the rhizosphere, a competitive and harsh environment, and as an endophyte capable of colonizing the internal plant tissues. *Micromonospora* species have less than 3% distance in their 16S rRNA genes, which can be roughly translated to a time of 150MY according to the equivalence proposed by Ochman and Wilson [89]. In the current phylogeny (Fig. 1), *M. lupini* has as closest neighbours *M. chokoriensis M. saelicesensis and M. zamorensis*, isolated from sandy soil, root nodules of *L. angustifolius* and the rhizosphere of *P. sativum* respectively. *Micromonospora* sp. L5 and *M. aurantiaca* are located further away, with a distance of 1.2% that would translate to 60 MY for the emergence of a group of species that interact with plants, a date that would be close to the postulated time of emergence of *Fabaceae* and that of many actinorhizal plant families [90]. The separation from the *Salinispora* and *Verrucosispora* lineages would constitute two independent events that would have occurred slightly earlier at 160MY and 170MY, while the emergence of the *Actinoplanes* and that of the *Dactylosporangium* would have occurred 250MY ago, a time when dicotyledons had not yet appeared but when continents and thus soils had appeared that did permit the growth of primitive plants such as the gymnosperms.

The size of the *Micromonospora* genomes analyzed is quite uniform, with that of Lupac 08 slightly larger. The chromosome size of *M. lupini* Lupac 08 appears to reflect a wealth of genes allowing for adaptation to a complex saprophytic/endophytic lifestyle, which means adapting to a wider range of environmental conditions with the ability to metabolize a large variety of nutrient sources. Considering that *Micromonospora* sp. L5 and *M. lupini* Lupac 08 were both isolated from nitrogen fixing nodules (actinorhizal and legume plants, respectively), it would be expected that the genomes of these strains be more similar to each other than to *M. aurantiaca* ATCC 27029^T^ which was originally isolated from soil. Surprisingly this was not the case as confirmed by the high number of strain specific genes identified in the genome of Lupac 08, suggesting a high capacity of adaptation to a fluctuating environment by this microorganism. On the other hand, *Micromonospora* sp. L5 and *M. aurantiaca* ATCC 27029^T^ share a high number of orthologous genes (86%) suggesting that the niche of origin is not crucial.

An interesting result was the distribution of the metabolic profiles of 20 bacteria representing different living environments (Fig. 5). There was a clear proximity between *M. lupini* Lupac 08 and the three *Frankia* genomes. This result suggests that strain Lupac 08 contains metabolic functions similar to those found in *Frankia* strains that are probably useful for its interaction with plants. This metabolic versatility combined with a diverse transport system make Lupac 08 an organism fit to adapt to a soil/plant environment.

The emergence and evolution of nitrogen fixation ability among the domains *Bacteria* and *Archaea* is complex and has not yet been fully elucidated. Although it was previously reported that *Micromonospora* strains isolated from legume and actinorhizal root nodules contained *nifH*-like gene fragments [11], [12], we could not confirm these results. In a similar approach based on PCR-amplification, other authors reported the presence of *nif*-H like sequences for bacterial isolates obtained from legumes collected in arid zones including *Microbacterium*, *Agromyces*, *Mycobacterium* and *Ornithinicoccus* [91]. One recurrent problem with the use of a PCR-based approach is that it is limited to a single gene amplified billions of times, which may provide false-positive results [92] and for this reason must always be confirmed by an independent approach.

Plant-polymer degrading enzymes such as cellulases and pectinases have been suspected to play a role in internal colonization. Most plant pathogens secrete cellulases, pectinases, xylanases, or other enzymes to hydrolyze plant cell wall polymers, while a lack of secreted hydrolases has been proposed to be favourable for microorganisms that form beneficial association with plants. Examples of endophytic plant growth promoting bacteria that lack large amounts of cell wall degrading enzymes include *Frankia* [38], *Enterobacter* sp. 638 [47], *Azoarcus* sp. BH72 [60] and *Herbaspirillum seropedicae* [93]. An *Azospirillum* sp. that does not colonize root tissues proper, but only the rhizosphere, has a genome containing a large number of putative cellulases similar to soil cellulolytic bacteria with 26–34 glycosyl hydrolases [94], as compared to the 37 present in *T. fusca*, a highly cellulolytic actinobacterium isolated from soil.

The genome of *M. lupini* revealed a high number of putative genes that encode for hydrolytic enzymes and specifically cellulolytic, xylanolytic, chitinolytic and pectinolytic activities were confirmed in the laboratory, indicating the capacity of *Micromonospora* to degrade plant polymers in a way similar to that of plant pathogen microorganisms. In the case of *Micromonospora*, there seems to be a paradox since strain *M. lupini* Lupac 08 shows a very high *in vitro* activity for cellulases and xylanases, however, preliminary inoculation experiments in our laboratory indicate that the microorganism does not behave as a pathogen, on the contrary, *Micromonospora* appears to interact in a tripartite relationship stimulating nodulation and plant growth (Fig. 7c). Therefore the question arises as to what is the function of these enzymes when *Micromonospora* interacts with the host plant. Alternatively some of the genes present, especially those related to the metabolism of cellulose may not necessarily imply that the bacterium is involved in plant cell wall degradation but have a different role, yet to be defined [95]. In addition many of the cellulose-related genes contain binding-domains suggesting that these may be related to the adhesion of the bacterium to the plant. These genes could also help *Micromonospora* digest plant cell walls upon senescence of the nodules.

*M. lupini* Lupac 08 contains several secondary metabolite gene clusters, many of which appear to be involved in the synthesis of siderophores and also of antibiotics. These would also in all likelihood be involved in the synthesis of the antitumor anthraquinone molecules described previously [9], [10]. *Micromonospora*, like many other endophytic bacteria is a facultative plant colonizer that must compete with other microorganisms in the rhizosphere before entering the plant. In this sense, the NRPS and PKS gene clusters identified in the genome of *M. lupini* Lupac 08 may be involved in defense as well as in interaction and communication with its host plant. Thus, it will be necessary to identify these compounds and their functional attributes to further expand our knowledge of this plant-microbe interaction.

## Conclusions

We have provided experimental data which supports the hypothesis that *M. lupini* Lupac 08 is a plant growth promoting bacterium. *Micromonospora lupini* Lupac 08 clearly produces a plant growth enhancing effect as observed in laboratory experiments. The localization of several genes involved in plant growth promotion traits such as the production of siderophores, phytohormones, the degradation of chitin (biocontrol) and the biosynthesis of trehalose may all contribute to the welfare of the host plant. *Micromonospora* appears as a new candidate in plant-microbe interactions with an important potential in agriculture and other biotechnological applications. The current data is promising but it is still too early to determine which specific roles are played by this microorganism in interactions with nitrogen fixing plants.

## Methods

### Genome sequencing, annotation and analysis

The genome sequence of *M. lupini* Lupac 08 was determined using the 454 FLX system and Titanium platform (454 Life Sciences) as previously reported [96]. Sequences were assembled into 50 contigs and four scaffolds ranging from 583 to 7,083,659 nucelotides using the MaGe (Magnifying Genomes) interface [97]. This Whole Genome Shotgun project has been deposited at European Nucleotide Archive under accession number NZ_CAIE00000000.01.

### 16S rRNA gene phylogeny

Sequences obtained from public databases (Genbank/EMBL) were manually aligned using clustal X software [98]. Phylogenetic distances were calculated with the Kimura 2-parameter model [99] and the tree topologies were inferred using the maximum-likelihood method [100]. All analyses were carried out using the MEGA5 program [101].

### Comparative genome analysis

Genome rearrangement of the *Micromonospora* strains *M. lupini* Lupac 08, *Micromonospora* sp. L5, *M. aurantiaca* ATCC 27029^T^ and *Micromonospora* sp. ATCC 39149 were carried out using MAUVE software [102]. The number of shared and unique genes present in the respective genomes were calculated and represented by a Venn-diagram using the EDGAR software [103]. Potential horizontally transferred genes were predicted using the “Region of Genomic Plasticity Finder” method implemented on the MicroScope platform. First we selected genomes included in the PkGDB and NCBI RefSeq databases that presented high synteny percentages with the Lupac 08 strain. Automatic results were manually curated according to several features such as base composition, DNA repetitions, presence of near mobile elements and information provided by SIGI and IVON programs [104].

### Comparative analysis of metabolic profiles

A bicluster plot of the metabolic profiles for *M lupini* Lupac 08 and 20 other bacterial genomes was performed with Multibiplot [105]. A comparison of 798 MicroCyc metabolic pathways was made using MaGe. This comparison is based on the calculation of ‘pathway completion’ values, scaled from 0 to 1, where 0 means that a particular organism does not contain any enzyme for a given pathway and 1 that it contains all the reactions of the pathway. These values were transformed applying row standardization and a JK-Biplot was constructed after performing a PCA (Singular Value Decomposition estimation method). The heatmap was then obtained with the expected values computing the Euclidian distance and average linkage for rows and columns.

### Transport proteins identification and classification

Information about transport proteins of genomes was obtained from the TransportDB relational database when available (http://www.membranetransport.org/). The identification and classification of the transporters of strain Lupac 08 was performed using the TransAAP tool based on TransportDB [106] followed by manual validation.

### Cellulose, starch and xylan degradation

Strain Lupac 08 was cultivated on yeast-malt agar for 5 days and subsequently transferred to M3 agar [107] with and without glucose and supplemented with one of the substrates in the following way: carboxymethylcellulose (CMC, 0.5%), starch (1%) and xylan (1%). A bacterial suspension of 10^6^ per ml was prepared in saline solution (0.85%) and 200 µl were inoculated on the different plates which were then incubated at 28°C and results were recorded at 4, 7 and 14 days after inoculation. Xylan and CMC plates were stained with Congo red [108] while starch plates were flooded with iodine solution [109].

### Pectin degradation

Pectinolytic acitivity was determined as described in Williams et al. [109]. Agar plates supplemented with pectin (0.5%) were streaked with strain Lupac 08 and incubated at 28°C. Hydrolysis zones were detected after 14 days incubation by flooding plates with an aqueous solution of cetyltrimethyl ammonium bromide (CTAB, 1%) and examining them after 30 min.

### Chitin degradation

Chitinolytic acitivity was determined as described in Murthy et al. [110]. Agar plates supplemented with colloidal chitin at 0.5% (Gift of France-Chitine, Orange, http://france-chitine.com/), partly hydrolysed by stirring in 0.5 M HCl for 2h, were inoculated with strain Lupac 08 and incubated at 28°C. Hydrolysis zones were detected as cleared zones after 14 days incubation.

### Siderophore production

Siderophore production was assessed using a modified chrome azurol S (CAS) assay [111]. Strain Lupac 08 was cultured on yeast-malt agar and incubated for 7 days, subsequently it was streaked onto CAS agar plates and incubated at 28°C for 7–10 days. A positive result was indicated by an orange halo around bacterial colonies.

### Indole-3-acetic acid production

Production of indole acetic acid was assayed following the method of Glickmann and Dessaux [112]. Strain Lupac 08 was inoculated in 5 ml of yeast-malt medium supplemented with L-tryptophan (0.2%) and incubated at 28°C at 150 rpm during 7 days. The culture was then centrifuged at 12,000 x g for 10 min and 1 ml of the supernatant was mixed with 2 ml of Salkowski’s reagent [113] and incubated at room temperature for 30 min. IAA production was measured spectrophotometrically at 530 nm to assess the development of a pink colour.

### Plant growth

Surface-sterilized seeds of *Trifolium* sp. were germinated axenically in Petri dishes on 1.4% w/v agar. Seedlings were transferred to sterile square plastic plates that contained a nitrogen-free nutrient solution [114]. Fifteen plantlets were inoculated in the following manner (5 per treatment): 500 µl (each) of bacterial suspensions (10^6^ cells per ml) of *M. lupini* Lupac 08 and *Rhizobium* sp. E11 for coinoculation treatment; inoculation with *Rhizobium* sp. E11; and uninoculated plants as negative controls.

### Acetylene reduction activity

Nitrogenase activity was measured using acetylene reduction [115] in sterile 150 ml plasma flasks with a rubber stopper. Cells of Lupac 08 were cultured in liquid minimal glucose medium without nitrogen at 28°C with shaking. The air in flasks was replaced with mixture of air and acetylene (ration 90:10 v/v). One mililiter of mixture was sampled for each measure using gas chromatography with a flame ionization detector (Girdel 30, France). *Mesorhizobium melitolti* Sm1021 was used as positive control.

## Supporting Information

Table S1 ***M. lupini*** **Lupac 08 genome distribution of 4873 CDS (70.2%) based on COG categories.**
(DOCX)

Table S2 **Carbohydrate related loci including cell-wall degrading enzymes and their potential regulators located on the genome of** ***M. lupini*** **Lupac 08.**
(DOCX)
