# Supplementary material for: Genome Features of the Endophytic Actinobacterium Micromonospora lupini Strain Lupac 08: On the Process of Adaptation to an Endophytic Life Style?
Source: PLoS One. 2014 Sep 30;9(9):e108522. doi: 10.1371/journal.pone.0108522 (PMC4182475; doi:10.1371/journal.pone.0108522)
Supplement: Table S1 — M. lupini Lupac 08 genome distribution of 4873 CDS (70.2%) based on COG categories. (DOCX) [file pone.0108522.s001.docx]

**Supplementary Table 1**. *M. lupini* Lupac 08 genome distribution of 4873CDS (70.2%) based on COG categories.

| **Class ID** | **Description** | **CDS** | **%** |
| --- | --- | --- | --- |
| D | Cell cycle control, cell division, chromosome partitioning | 59 | 0.84 |
| M | Cell wall/membrane/envelope biogenesis | 284 | 4.02 |
| N | Cell motility | 61 | 0.86 |
| O | Posttranslational modification, protein turnover, chaperones | 190 | 2.69 |
| T | Signal transduction mechanisms | 387 | 5.48 |
| U | Intracellular trafficking, secretion, and vesicular transport | 66 | 0.93 |
| V | Defense mechanisms | 182 | 2.58 |
| W | Extracellular structures | 4 | 0.06 |
| Z | Cytoskeleton | 1 | 0.01 |
| A | RNA processing and modification | 1 | 0.01 |
| B | Chromatin structure and dynamics | 2 | 0.03 |
| J | Translation, ribosomal structure and biogenesis | 248 | 3.51 |
| K | Transcription | 763 | 10.81 |
| L | Replication, recombination and repair | 283 | 4.01 |
| C | Energy production and conversion | 390 | 5.52 |
| E | Amino acid transport and metabolism | 903 | 12.79 |
| F | Nucleotide transport and metabolism | 135 | 1.91 |
| G | Carbohydrate transport and metabolism | 685 | 9.70 |
| H | Coenzyme transport and metabolism | 214 | 3.03 |
| I | Lipid transport and metabolism | 308 | 4.36 |
| P | Inorganic ion transport and metabolism | 612 | 8.67 |
| Q | Secondary metabolites biosynthesis, transport and catabolism | 286 | 4.05 |
| R | General function prediction only | 1251 | 17.72 |
| S | Function unknown | 356 | 5.04 |
