# Supplementary material for: Genome Features of the Endophytic Actinobacterium Micromonospora lupini Strain Lupac 08: On the Process of Adaptation to an Endophytic Life Style?
Source: PLoS One. 2014 Sep 30;9(9):e108522. doi: 10.1371/journal.pone.0108522 (PMC4182475; doi:10.1371/journal.pone.0108522)
Supplement: Table S2 — Carbohydrate related loci including cell-wall degrading enzymes and their potential regulators located on the genome of M. lupini Lupac 08. (DOCX) [file pone.0108522.s002.docx]

**Table S2**. Carbohydrate related loci including cell-wall degrading enzymes and potential regulators located in the genome of *M. lupini* Lupac 08.

| **Code** | **Locus Tag: MILUP08v1_** | |  | **Type** | **Family** | **Localization** | **Regulator** | **Locus Tag: MILUP08v1_** |
| --- | --- | --- | --- | --- | --- | --- | --- | --- |
| **CELLULOSE RELATED PROTEINS** | | | | |  |  |  |  |
| C1 | 40696 | | celA | Endoglucanase A (cellobiohydrolase) | GH6 | Secreted | TR, AraC family | 40694 |
| C2 | 41235 | | cel | Secreted endo-1,4-β-glucanase | GH12 | Secreted | - | - |
| C3 | 41640 | | celA | Extracellular cellulase | GH5; CBM2 | Secreted | - | - |
| C4 | 41767 | | bglB | β-glucosidase B | GH1 |  | celR ABC transporter sugar-binding protein/LacI TR | 41768 |
| C5 | 41776 | | celB | Extracellular cellulase B | GH9 | Secreted | TR, MocR | 41774 |
| C6 | 41902 | | celA | Extracellular cellulase | GH44; CBM2 | Secreted | TR, TraR/DksA | 41904 |
| C7 | 42012 | | cel | Extracellular cellulase | CBM2; CBM49 | Secreted | TR, LacI family * | 42015 |
| C8 | 42027 | | faeC | Extracellular ferruloyl esterase, hemicellulase | CBM2 | Secreted | - |  |
| C9 | 42309 | | cel | Extracellular cellulase 1 | GH9; CBM4-9; CBM2 |  | TR, XRE family ** | 42313 |
| C10 | 42938 | | - | Extracellular cellulose-binding endoglucanase (fragment) | CBM2 | Secreted |  |  |
| C11 | 42941 | | - | Extracellular cellulose-binding endoglucanase | CBM2 | Secreted | Two-component RR containing a CheY-like receiver | 42945 |
| C12 | 44196 | | celA (celE) | Extracellular cellulase (Endoglucanase E-5) | GH5; CBM2 | Secreted | - |  |
| C13 | 44350 | | celD | Extracellular cellulase E-4 | CBM2 |  | TR, LacI | 44353 |
| C14 | 44540 | | faeC | Extracellular ferruloyl esterase; hemicellulase | ricinB lectin | Secreted | - |  |
| C15 | 44893 | | CelA | Extracellular endoglucanase (Chitin-binding domain 3) | CBM2, ChB3 | Secreted | - |  |
| C16 | 45863 | | cbhA | Extracellular Exoglucanase A, 1, 4-beta cellobiohydrolase | GH6 | Secreted | - |  |
| C17 | 30016 | | abfB | α-L-arabinofuranosidase, cellulose-binding domain | CBM2; GH62 | Secreted | Two-component RR receiver | 30017 |
| C18 | 40087 | | - | Cellulose-binding surface protein | CBM2 | Membrane | TR, SARP family* | 40090 |
| C19 | 41639 | | pel | Extracellular pectate lyase A (modular protein); cellulose-binding | CBM2; | Secreted | - |  |
| C20 | 41828 | | - | Glycoside hydrolase family 5 with Cellulose-binding domain | Cellulase glycosyl hydrolase family 5; CBM2 | Membrane-bound | - |  |
| C21 | 41918 | | celD | Cellulose-binding family II | CBM2 | Secreted | Two-component system sensor histidine kinase* | 41923 |
| C22 | 41935 | | - | Cellulose-binding surface protein | CBM2 | Membrane-bound | TR, MarR/EmrR family* | 41938 |
| C23 | 41962 | | - | Feruloyl esterase C (modular protein); cellulose-binding | CBM2 | Secreted | TR, AfsR family | 41961 |
| C24 | 41963 | | - | Cellulose-binding family II (fragment) | CBM2 | Membrane-bound | TR, AfsR family | 41961 |
| C25 | 42792 | | - | Secreted cellulose-binding, family II, bacterial type | CBM2 | Secreted structure | - |  |
| C26 | 42910 | | - | Esterase with cellulose-binding domain | CBM2, CBM49 | Secreted | Two-component TR, LuxR family | 42913 |
| C27 | 42911 | | - | Esterase with cellulose-binding domain | CBM2 | Secreted | Two-component TR, LuxR family | 42913 |
| C28 | 42938 | | - | Extracellular endoglucanase (fragment) | CBM2 | Cell adhesion | - |  |
| C29 | 42941 | | - | Extracellular cellulose-binding endoglucanase | CBM2 | Cell adhesion | - |  |
| C30 | 42969 | | - | Secreted cellulose binding, type IV with ricinB domain | CBM6, ricin b lectin | Cell adhesion | - |  |
| C31 | 43377 | | - | Extracellular cellulose-binding, glucose/sorbosone dehydrogenase | CBM6 | Secreted | - |  |
| C32 | 43769 | | - | Exported protein of unknown function (cellulose-binding domain) | CBM2 | Secreted | - |  |
| C33 | 44017 | | - | Extracellular cellulose-binding hydrolase | CBM2 | Cell adhesion | TR, LysR family* and putative TR, MarR family | 44014 and 44020 |
| C34 | 44220 | | - | Cellulose-binding protein | GH9 | Secreted | Two-component RR | 44218 |
| C35 | 44223 | | - | Esterase (Cellulose-binding domain) | CBM2 | Secreted | - |  |
| C36 | 44233 | | - | Cellulose 1,4-beta-cellobiosidase | CBM2 | Secreted | TR, LacI family | 44231 |
| C37 | 44377 | | - | Cellulose binding secreted glycosyl hydrolase | CBM6, GH11, GH43 | Secreted; scavange | Two-component system RR and TR, LysR family | 44375 and 44379 |
| C38 | 44479 | | - | Putative glycoside hydrolase (cellulose-binding family II protein) | GH5 | Secreted | - |  |
| C39 | 44603 | | - | Conserved protein of unknown function (secreted cellulose-binding protein) | CB3 | Secreted | - |  |
| C40 | 44755 | | - | Protein of unknown function (cellulose-binding domain) | CBM2 | Secreted | Putative TR ** | 44749 |
| C41 | 44788 | | - | Glycoside hydrolase, cellulose-binding family II | GH48 | Secreted | - |  |
| C42 | 45021 | | - | Cellulose-binding protein | CBM2, CelA multidomain | Secreted | TR, AsnC family and TR Rrf2 family ** | 45019 and 45024 |
| C43 | 45309 | | - | Extracellular cellulose-binding glycoside hydrolase | CBM6, GH31 | Secreted | TR, XRE family ** | 45313 |
| C44 | 45318 | |  | Extracellular cellulose-binding peptidase | CBM2 | Secreted | TR, AfsR family | 45319 |
| C45 | 45357 | | - | Extracellular cellulose-binding xylosidase | GH43, ricin B lectin domain | Secreted | TR, GntR family * | 45360 |
| C46 | 46280 | | - | Extracellular cellulose-binding protein with PKD domains | CBM6_xyl, ThuA, PKD | Membrane-bound | - |  |
| **XYLAN RELATED PROTEINS** | | | | |  |  |  |  |
| X1 | 41153 | | xlnA | Extracellular xylanase | SGNH hydrolase; B ricin B lectin | Secreted | - |  |
| X2 | 41779 | | xlnA | Extracellular xylanase-arabinofuranosidase bifunctional enzyme | GH62, GH10 | Secreted | - |  |
| X3 | 42956 | | xlnA | Extracellular endo-1,4-beta-xylanase | GH43 | Secreted | TR, GntR family * | 42959 |
| X4 | 42964 | | - | Putative xylanase A | - | Secreted | Binding-protein-dependent ABC transport system | 42961 |
| X5 | 42975 | | xlnA | Extracellular Endo-1,4-beta-xylanase with a ricin B domain | Ricin B domain | Secreted | TR, LacI family | 42979 |
| X6 | 42986 | | xlnA | Extracellular Endo-1,4-beta-xylanase B | GH10 | Secreted | TR LacI; TR GntR family ** | 42986 and 42990 |
| X7 | 43449 | | xlnA | Extracellular endo-1,4-beta-xylanase | GH10 | Secreted | - |  |
| X8 | 44411 | | - | Extracellular bifunctional xylanase/deacetylase [Includes: Endo-1,4-beta-xylanase D ; Acetylated xylan deacetylase] | CBM2; CE4 | Secreted | - |  |
| X9 | 44441 | | xlnA | Extracellular endo-1,4-beta-xylanase B | GH11; CBM2 | Secreted | - |  |
| X10 | 44484 | | xlnA | Extracellular endo-1,4-beta-xylanase A | GH10; ricinB lectin | Secreted | - |  |
| X11 | 44601 | | xlnA | Extracellular endo-1,4-beta-xylanase (with Cellulose-binding domain) | GH62; GH10 | Secreted | Putative TR, TetR family ** | 44597 |
| X12 | 44692 | | xlnA | Extracellular endo-1,4-beta-xylanase A | ricinB lectin | Secreted | TR, LacI family ** | 44696 |
| **CHITIN RELATED PROTEINS** | | | | |  |  |  |  |
| CH1 | 41110 | | - | Chitin-binding domain 3 protein | related to cellulose-binding | Secreted | - |  |
| CH2 | 41724 | | - | Chitin-binding domain 3 | CB-D3 ,CBM2 | Membrane-bound | - |  |
| CH3 | 41729 | | cbp | Chitin-binding domain 3 protein | CBD3; Chi1A-BD | Membrane-bound | - |  |
| CH4 | 41789 | | cbp | Polysaccharide/chitin/xylan deacetylase; Chitooligosaccharide deacetylase | CE4-nodB like; CE4-SF | Secreted | Two-component system RR receiver protein | 41786 |
| CH5 | 41912 | | ChiD | Extracellular endochitinase D | GH18-chitinase | Secreted | - |  |
| CH6 | 43481 | | chiA | Extracellular chitinase | GH18-chitinase-D | Secreted | TR, HxlR family; TR, AsnC family * | 43481 and 43484 |
| CH7 | 44343 | | chiC | Extracellular chitinase II | GH18-chitinase | Secreted | TR, XRE family ** | 44347 |
| CH8 | 45172 | | chiC | Extracellular chitinase C | GH18-chitinase | Secreted | - |  |
| CH9 | 45568 | | hex | Beta-N-acetylhexosaminidase | GH20-SpHex-like | - | TR, MarR family ** | 45564 |
| **PECTIN RELATED PROTEINS** | | | | |  |  |  |  |
| P1 | 41227 | | pel | Pectate lyase | Laminin G3, | - |  |  |
| P2 | 42909 | | pmeA | Extracellular pectin methylesterase | Putative pectinesterase | Secreted | Two component, TR | 42908 |
| P3 | 43780 | | - | Putative pectin lyase | pectate-lyase-3 | - | TR, AsnC family ** | 43784 |
| P4 | 44473 | | pel | Pectate lyase | Pec_lyase_C superfamily | - | - |  |
| P5 | 44896 | | - | Putative Pectin lyase fold/virulence factor | - | Secreted | - |  |
| P6 | 44897 | | - | Exported protein of unknown function; putative Pectin lyase fold/virulence factor | No putative conserved domains detected | Secreted | - |  |
| P7 | 43920 | | pel | Pectate lyase (: D-galacturonate catabolism) | Pec_lyase_C superfamily | Secreted | TR, SARP family | 43922 |
| **MISCELLANEOUS PROTEINS** | | | | |  |  |  |  |
| M1 | | 40202 | - | Polyssacharide deacetylase | Catalytic NodB homology domain of rhizobial NodB-like proteins | - | TR, Mer Family | 40203 |
| M2 | | 40215 | aglA | α-glucosidase | Trehalose Tre_C | - | TR, LacI family | 40211 |
| M3 | | 40216 | - | Mycodextranase | CBM6; discoidin-1 | - | - |  |
| M4 | | 40218 | - | Secreted glycosyl hydrolase | CBM6; discoidin-1 | Secreted | - |  |
| M5 | | 40490 | - | Exported protein of unknown function | CBM5/12 | Secreted | Putative TetR-family TR | 40493 |
| M6 | | 40533 | - | Putative glycosyl hydrolase or sugar phosphorilase | GH59 | - | - |  |
| M7 | | 40618 | - | Extracellular sugar-binding protein family 1 | SBP_Bac_1 | - | dgoR galactonate operon transcriptional repressor | 40616 |
| M8 | | 40621 | - | Alpha-galactosidase C | GH37 | - | - |  |
| M9 | | 40687 | - | Galactosecerebrosidase | GH59 | - | Two-component TR, LuxR family | 40690 |
| M10 | | 40688 | - | Putative ferruloyl esterase | Ricin_B_lectin | - | Two-component TR, LuxR family | 40690 |
| M11 | | 40718 | - | Secreted Ricin B-related lectin | Ricin_B_lectin | Secreted | TR, SARP family | 40714 |
| M12 | | 40720 | - | Secreted Ricin B-related lectin | Ricin_B_lectin | Secreted | - |  |
| M13 | | 40721 | - | β-galactosidase; Carbohydrate-binding ricinB domains | GH2 |  | - |  |
| M14 | | 40854 | - | α-glucosidase | GH42 |  | TR, LacI family | 40854 |
| M15 | | 40856 | malE | malE extracellular maltose-binding protein family 1 | SBP_Bac_1 | Secreted | TR, LacI family | 40854 |
| M16 | | 40932 | - | Lysozyme M1 | GH25 |  | - |  |
| M17 | | 41019 | mshB | D-myo-inositol 2-acetamido-2-deoxy-alpha-D-glucopyranoside deacetylase | CE14 |  | - |  |
| M18 | | 41122 | - | Ricin B lectin | Ricin_B_lectin |  | TR, AraC family | 41120 |
| M19 | | 41154 | - | Putative hydrolase with carbohydrate-binding domain | CBM 2 |  | - |  |
| M20 | | 41226 | - | Glycoside hydrolase | GH15 |  | - |  |
| M21 | | 41304 | - | Glycoside hydrolase family 3 domain protein | GH 3 |  | - |  |
| M22 | | 41305 | - | Carbohydrate-binding protein | Ricin_B_lectin |  | - |  |
| M23 | | 41313 | - | Glycoside hydrolase 15-related protein | GH15 |  | - |  |
| M24 | | 41324 | - | Glycosidase hydrolase family 3 domain protein | GH3 |  | TR, ROK family | 41325 |
| M25 | | 41370 | manB | β-mannosidase | GH2 |  | ABC transporter sugar-binding protein/LacI TR | 41369 |
| M26 | | 41572 | - | Amylo-α -1,6-glucosidase | GDB1 |  | ABC transporter sugar-binding protein/LacI TR | 41573 |
| M27 | | 41728 | - | Conserved protein of unknown function | CBM48 |  | - |  |
| M28 | | 41764 | - | Extracellular sugar-binding protein family 1 | SBP_1 |  | - |  |
| M29 | | 41833 | galA | α-galactosidase | GH 37 |  | - |  |
| M30 | | 41836 | - | Extracellular solute-binding protein family 1 | SBP1 |  | TR, DeoR_family | 41838 |
| M31 | | 41837 | galB | β-galactosidase 1 | GH42 |  | TR, DeoR_family | 41838 |
| M32 | | 41959 | - | Glycogen debranching enzyme; Amylo-α -1,6-glucosidase | GDE_C; GDB1 |  | TR, AfsR_family | 41961 |
| M33 | | 41964 | abfB | α-L-arabinofuranosidase | GH62 |  | TR, AfsR family | 41961 |
| M34 | | 42010 | xynB | Β-xylosidase; α-N-arabinofuranosidase | GH43 |  | ABC transporter sugar-binding protein/LacI TR | 42015 |
| M35 | | 42019 | - | Extracellular sugar-binding protein | - |  | TR,TetR family | 42023 |
| M36 | | 42020 | xlnA | Extracellular beta-xylosidase | Ricin B lectin |  | TR, TetR family | 42023 |
| M37 | | 42022 | abfB | α-L-arabinofuranosidase | CBM 13 |  | TR, TetR family | 42023 |
| M38 | | 42028 | - | Galactocerebrosidase | GH59 |  | - |  |
| M39 | | 42029 | abfA | α-L-arabinofuranosidase B catalytic | CBM2 |  | (-)TR, XRE family | 42038 |
| M40 | | 42043 | mshB | 1D-myo-inositol 2-acetamido-2-deoxy-α -D-glucopyranoside deacetylase | - |  | Two component TR, LuxR_family | 42042 |
| M41 | | 42075 | - | β-xylosidase | Ricin B lectin |  | Sugar-binding ABC transporter/LacI TR | 42076 |
| M42 | | 42095 | - | Glycoside hydrolase family 16 with Ricin B domains | GH 16, CBM 13 |  |  |  |
| M43 | | 42162 | - | Amylo-α-1,6-glucosidase (glycogen debranching enzyme) | GDB1 |  | TR, TetR family | 42156 |
| M44 | | 42219 | - | 1,4-α-glucan-branching enzyme | CBM 48 | - | TR, AraC family | 42118 |
| M45 | | 42220 | glgE | Glucanase glgE | DUF 3416 | Cytoplasmic | TR, AraC family | 42118 |
| M46 | | 42330 | - | Glycoside hydrolase | GH 15 | - | TR, HxlR family | 42327 |
| M47 | | 42369 | - | Glycoside hydrolase family 3 domain protein | GH 3 | - | - | - |
| M48 | | 42432 | - | Glycoside hydrolase clan GH-D (modular protein) | Ricin B lectin | Secreted | - | - |
| M49 | | 42497 | - | Exported glycoside hydrolase | GH 26 | Secreted | - | - |
| M50 | | 42794 | - | Extracellular glycoside hydrolase | CBF 6 | Secreted | TR, TetR family | 42797 |
| M51 | | 42779 | - | Galactosidase | GH 2 | - | - | - |
| M52 | | 42781 | - | Galactosidase | - | - | - | - |
| M53 | | 42839 | - | Glycoside hydrolase | GH 15 | - | - | - |
| M54 | | 42868 | abfA | α-N-arabinofuranosidase | GH 43 | Secreted | TR, GntR family with aminotransferase domain | 42872 |
| M55 | | 42976 | - | Putative hydrolase with a ricinB domain | Ricin superfamily | Secreted | TR, LacI family | 42979 |
| M56 | | 42977 | agaA | α-galactosidase | Ricin B lectin | - | TR, LacI family | 42979 |
| M58 | | 42978 | - | Glycosyl hydrolase | GH 32 | - | TR, LacI family | 42979 |
| M59 | | 42983 | - | Carboxylesterase | β-lactamase superfamily | - | RR with ANTAR domains | 42984 |
| M60 | | 43068 | - | Acetyl xylan esterase | - | - | TR, LacI family | 43067 |
| M61 | | 43072 | - | Secreted carbohydrate binding protein | - | Secreted | - |  |
| M62 | | 43105 | - | Transglycosylase with peptidoglycan-binding lysM domains | LysM superfamily | Membrane-bound | Signal transduction response regulator, SARP family | 43102 |
| M63 | | 43195 | - | Amylo-alpha-1,6-glucosidase (glycogen debranching enzyme) | GDB1 | Cytoplasmic | TR, LacI family | 43192 |
| M64 | | 43224 | treX | Glycogen debranching enzyme | GH 13, CBM 48 | - | - |  |
| M65 | | 43226 | treZ | Malto-oligosyltrehalose trehalohydrolase | GH 13, CBM 48 | - | - |  |
| M66 | | 43269 | glgA | Amylo-alpha-1,6-glucosidase; glycogen synthase | GDB1 | Cytoplasmic | TR, MarR family | 43268 |
| M67 | | 43466 | - | Endo-1,3(4)-beta-glucanase | GH 81 | Secreted | TR, XRE family | 43463 |
| M68 | | 43489 | - | Polysaccharide deacetylase | CE 4 | - | TR, TetR family | 43491 |
| M69 | | 43737 | abnA | Glycoside hydrolase family 43 COG3507 β-xylosidase Probable arabinan endo-1,5-α -L-arabinosidase A | GH 43 | - | TR, ArsR family | 43736 |
| M70 | | 43912 | - | Extracellular sugar-binding protein | SBP 1 | Membrane-bound | TR, LacI family | 43913 |
| M71 | | 43976 | - | Conserved secreted protein of unknown function (Galactose-binding domain-like) | - | Secreted | Putative ROK family TR | 43977 |
| M72 | | 43982 | bglA | β-glucosidase A | GH 1 | Cytoplasmic | ArsR family TR | 43986 |
| M73 | | 44031 | - | Conserved exported protein of unknown function (Carbohydrate Binding) | - | - | Putative TR, TetR family | - |
| M74 | | 44170 | - | Protein of unknown function; Galactose-binding domain | - | - | - | - |
| M75 | | 44230 | - | 1,4-β-xylosidase | GH 3 | Cytoplasmic | TR, LacI family | 44231 |
| M76 | | 44232 | - | Acetyl xylan esterase | AXE 1 | - | TR, LacI family | 44231 |
| M77 | | 44351 | - | Esterase | PHB depolymerase family | Secreted | TR, LacI family | 44353 |
| M78 | | 44357 | xynB | α-L-arabinofuranosidase | Putative GH | - | TR, LysR family | - |
| M79 | | 44360 | - | Conserved secreted protein of unknown function; putative ricinB domains | - | - | TR, LysR family | 44359 |
| M80 | | 44361 | - | Chitosanase | GH 46 | Secreted | TR, MerR family | - |
| M81 | | 44432 | - | Secreted esterase | PHB depolymerase family | Secreted | paaX Phenylacetic acid-responsive TR | 44430 |
| M82 | | 44457 | aguA | α-glucuronidase | GH 67 | - | TR, LacI family | - |
| M83 | | 44498 | - | Conserved protein of unknown function (DGPF domain) | CBM 57 | - | - | - |
| M84 | | 44515 | xynC | Extracellular glycoside hydrolase with carbohydrate-binding domain | GH 30 | Secreted | TetR family TR | 44518 |
| M85 | | 44524 | - | Putative glycosyl hydrolase (Galactose-binding domain) | GH 2 | Secreted | Putative TR | 44522 |
| M86 | | 44538 | - | Glycoside hydrolase with ricin domain | GH 5 | - | - | - |
| M87 | | 44539 | abfB | α-N-arabinofuranosidase B | GH 43 | Secreted | - | - |
| M88 | | 44542 | ugl | Unsaturated glucuronyl hydrolase | GH | - | - |  |
| M89 | | 44565 | abfA | α-L-arabinofuranosidase | - | Cytoplasmic | TR, LacI family | 44564 |
| M90 | | 44651 | - | Putative lysozyme (modular protein) | - | - | Putative two-component system RR | 44650 |
| M91 | | 44672 | - | Membrane protein of unknown function; putative Glycoside hydrolase domain | - | - | - |  |
| M92 | | 44694 | lacZ | β-galactosidase | GH 2 | Cytoplasmic | TR, LacI family | 44696 |
| M93 | | 44762 | bgaM | Extracellular β-galactosidase, ricinB domains | - | - | Putative TR | 44759 |
| M94 | | 44789 | bglA | β-glucosidase | GH 1 | Cytoplasmic | - | - |
| M95 | | 44831 | xylS | α-xylosidase | GH 31 | - | - | - |
| M96 | | 44832 | bglA | β-glucosidase A | GH 1 | Cytoplasmic | - | - |
| M97 | | 44871 | nagD | N-acetylglucosamine-6-phosphate deacetylase | CE 9 | - | - | - |
| M98 | | 44898 | amyE | Extracellular α-amylase | GH 13; CBM 41, 48 | Secreted | - | - |
| M100 | | 44900 | - | Carbohydrate-binding protein | CBM 2 | - | - | - |
| M101 | | 44901 | - | Carbohydrate-binding (similar to cellulose binding family II) | - | - | - | - |
| M102 | | 45151 | - | Extracellular sugar-binding component of ABC-type transport system | - | - | - | - |
| M103 | | 45258 | phaZ | Poly(3-hydroxyalkanoate) depolymerase C | - | - | - | - |
| M104 | | 45373 | bglB | Thermostable β-glucosidase B | GH 1 | Cytoplasmic | TR, LysR family* | 45369 |
| M105 | | 45388 | - | Glycoside hydrolase family 3 | GH 3 | - | - |  |
| M106 | | 45405 | - | Endo-1,4- β-glucanase/xyloglucanase | CBM 2 | Secreted | ATP-dependent TR, MalT-like, LuxR family | 45409 |
| M107 | | 45421 | amyE | Extracellular α-amylase | - | Secreted | Two component TR, LuxR family | 45423 |
| M108 | | 45524 | glcI | Glucan endo-1,3-β-glucosidase | GH | Membrane-bound | Two-component system RR | 45522 |
| M109 | | 45525 | glcI | Glucan endo-1,3-β-glucosidase |  |  | Two-component system RR | 45522 |
| M110 | | 45636 | - | Extracellular xylan 1,4-beta-xylosidase | Ricin superfamily | Secreted | - | - |
| M111 | | 45803 | - | Amylo-α-1,6-glucosidase, glycogen debranching enzyme | Amylo-α-16 | Cytoplasmic | Two-component RR receiver, LuxR family | 45800 |
| M112 | | 45837 | - | Extracellular ABC-type sugar-binding protein | - | - | TR, LacI family | 45840 |
| M113 | | 45841 | agl | α-glucosidase | AmyA | - | TR, LacI family | 45840 |
| M114 | | 45935 | - | Polysaccharide deacetylase | CE 4 | - | - | - |
| M115M116 | | 46103 | mshB | 1D-myo-inositol 2-acetamido-2-deoxy-alpha-D-glucopyranoside deacetylase | CE 14 | - | - | -- |
| M117 | | 46409 | - | Putative serine/threonine protein kinase (modular protein) | CBM 2 | - | - |  |
| M118 | | 46441 | - | α-L-arabinofuranosidase | - | Cytoplasmic | TR, LacI family | 46440 |
| M119 | | 46509 | - | Extracellular sugar-binding protein family 1 | - | - | - | - |
| M120 | | 46512 | bglB | Thermostable β-glucosidase B | GH 1 | Cytoplasmic | CelR TR, LacI family | 46513 |
| M121 | | 46537 | - | Amylo-alpha-16-glucosidase (fragment) | - | - | Two-component TR, winged helix family | 46539 |
| M122 | | 46548 | - | Polysaccharide deacetylase | CE 4 | - | Two-component system RR | 46548 |
| M123 | | 46592 | - | Putative lipolytic protein G-D-S-L family | - | - | Putative TR, ArsR family | 46590 |
| M124 | | 46619 | - | N-acetylglucosamine-6-phosphate deacetylase | - | Cytoplasmic | TR, DeoR family | 46616 |
| M125 | | 46824 | - | Carboxylesterase | COesterase | - | TR, GntR family; TR, TetR family | 46825 and 46826 |

CBD: Carbohydrate binding domain; SBP, solute binding protein; Bacterial extracellular solute-binding protein; GDB, glycogen debranching enzyme; DUF Domain of uknown function; AXE, acetyl xylane esterase; CC, crystalline cellulose; CB: Chitin-binding; CBM, carbohydrate binding module; CE, carbohydrate esterase; ChiA1, Chiting binding domain of CHiA1 proteins; SF, super family; CBP, chitin binding protein; GH glycoside hydrolase; CBF Carbohydrate binding family.

TR: Transcriptional Regulator; RR: Response Regulator.
